# Structure–activity relationships and cellular mechanism of action of small molecules that enhance the delivery of oligonucleotides

**DOI:** 10.1093/nar/gkx1320

**Published:** 2018-01-18

**Authors:** Rudolph L Juliano, Ling Wang, Francis Tavares, Edward G Brown, Lindsey James, Yamuna Ariyarathna, Xin Ming, Chengqiong Mao, Mark Suto

**Affiliations:** 1 Initos Pharmaceuticals LLC, Chapel Hill, NC 27599, USA; 2 UNC Eshelman School of Pharmacy, Chapel Hill, NC 27599, USA; 3 Chemogenics Biopharma, Research Triangle Park, NC 27709, USA; 4 Department of Cancer Biology and Comprehensive Cancer Center, Wake Forest University School of Medicine, Winston-Salem, NC 27157, USA; 5 Southern Research Institute, Birmingham, AL 35205, USA

## Abstract

The pharmacological effects of antisense and siRNA oligonucleotides are hindered by the tendency of these molecules to become entrapped in endomembrane compartments thus failing to reach their targets in the cytosol or nucleus. We have previously used high throughput screening to identify small molecules that enhance the escape of oligonucleotides from intracellular membrane compartments and have termed such molecules OECs (oligonucleotide enhancing compounds). Here, we report on the structure–activity relationships of a family of OECs that are analogs of a hit that emerged from our original screen. These studies demonstrate key roles for the lipophilic aromatic groups, the tertiary nitrogen, and the carbamate moiety of the parent compound. We have also investigated the intracellular site of action of the OECs and have shown that activity is due to the release of oligonucleotides from intermediate endosomal compartments rather than from early endosomes or from highly acidic downstream compartments. At high concentrations of OECs toxicity occurs in a manner that is independent of caspases or of lysosomal cathepsins but instead involves increased plasma membrane permeability. Thus, in addition to describing specific characteristics of this family of OECs, the current study provides insights into basic mechanisms of oligonucleotide trafficking and their implications for oligonucleotide delivery.

## INTRODUCTION

The precise gene regulating actions of siRNA, antisense oligonucleotides (ASOs), and splice switching oligonucleotides (SSOs) offer substantial promise for the therapy of cancer and other diseases (1–3). There have been massive investments in the chemistry and formulation of these molecules resulting in some promising advances in the clinic (4–8). However, use of oligonucleotides in therapy has been constrained by the inability to effectively deliver these relatively large and highly polar molecules (9,10). An important aspect of the delivery problem concerns trapping of oligonucleotides in endosomal compartments thus limiting access to their intracellular sites of action in the cytosol or nucleus (11–13).

Whether administered as ‘free’ molecules, as targeted conjugates, or associated with a nanocarrier, oligonucleotides enter cells by various endocytotic pathways (14). Initial internalization is followed by trafficking into multiple membrane-bound compartments including early/sorting endosomes, multi-vesicular bodies, late endosomes, lysosomes and the Golgi complex (15,16). During trafficking events, discontinuities in the lipid bilayer can occasionally occur (17), allowing partial escape of vesicle contents (18), and thus permitting the pharmacological effects of oligonucleotides. However, the escape process is usually rather inefficient. Intracellular trafficking involves numerous proteins and lipids that regulate the formation, movement and coalescence of membrane bound vesicles (19,20). Thus small organic molecules could potentially influence various aspects of intracellular trafficking and endomembrane stability, but few such molecules have been described thus far (21).

Based on these concepts, we previously undertook high throughput screening (HTS) of chemical libraries to seek small molecules that could enhance the pharmacological activities of oligonucleotides. In a prior publication we described the screening process and the initial characterization of a ‘hit’ from that screen, a molecule designated UNC10217938 (22). As we anticipated, this oligonucleotide enhancing compound (OEC) acted by promoting the release of oligonucleotides from endomembrane compartments thus increasing their access to RNA targets in the cytosol or nucleus. Other studies have also used high throughput screening and have found a variety of compounds, acting by several mechanisms, that improve the activities of oligonucleotides (23–25).

Here, we further pursue this approach and the development of UNC10217938 as an OEC via the synthesis and characterization of a number of analogs of this compound. We have systematically varied key moieties on the molecule and have established important structure activity relationships for this family of OECs. We have also investigated their mechanism of action at the subcellular level as well as the basis of their cytotoxicity. We show that effective but non-toxic concentrations of the OECs act after the early endosome stage but still at an upstream stage of the trafficking process rather than on late stage low pH compartments such as lysosomes. We also demonstrate that high concentrations of OECs cause cell death by a caspase-independent mechanism that involves changes in plasma membrane permeability. Although the OECs described herein are still at an early stage of development, our results suggest that some of these novel molecules may have promise as tools for understanding the intracellular behavior of oligonucleotides and their delivery systems, and as adjuncts for oligonucleotide delivery and therapeutics.

## MATERIALS AND METHODS

### Reagents, cells, and culture methods

HeLa Luc705 cells are stably transfected with a firefly luciferase reporter whose coding sequence is interrupted by an abnormal intron. Effective delivery of an appropriate SSO, such as the 2′-O-Me phosphorothioate SSO623 (5′-GTTATTCTTTAGAATGGTGC-3′), to the nucleus of these cells will correct splicing and allow luciferase expression (26). SSO623 was synthesized locally or purchased from Avecia. A 2′-O-Me gapmer phosphorothioate anti-MDR1 antisense oligonucleotide (5′-CCATCccgacctcgcGCTCC-3′) [2′-O-Me modifications in capitals] was from Integrated DNA Technologies. Lysotracker Red^®^ and Hoechst 33342 were obtained from ThermoFisher. EGA (4-bromobenzaldehyde *N*-(2,6-dimethylphenyl)semicarbazone) was purchased from Calbiochem. The mitochondrial selective dye TMRE (tetramethylrhodamine ethyl ester) was purchased from Abcam. CellLights 2.0 baculovirus vectors were bought from ThermoFisher. The pan-caspase inhibitor QVD-O-Ph (Quinoline-Val-Asp-Difluorophenoxymethyl Ketone) was from Cayman Chemicals while the cathepsin inhibitor E64d (2*S*,3*S-trans*-(ethoxycarbonyloxirane-2-carbonyl)-l-leucine-(3-methylbutyl)amide) was from Santa Cruz Biotechnology. A LIVE/DEAD^®^ cytotoxicity assay kit containing Calcein-AM and ethidium dimer was purchased from ThermoFisher.

### Synthesis of analogs

The synthesis and chemical characterization of the various analogs of UNC10217938 (abbreviated henceforth as UNC7938) are fully described in the Supplementary Information. All compounds were purified by liquid chromatography and characterized by nuclear magnetic resonance analysis and LC–MS. Compounds synthesized at the UNC Eshelman School of Pharmacy are designated by the prefix UNC and a four-digit identifier, for example UNC5103. Compounds synthesized by Chemogenics Biopharma of Research Triangle Park, NC are designated by the prefix B and a two-to four-digit identifier, for example B-48. Compounds UNC10217938, UNC10244093 and UNC10244102 were originally provided by Southern Research Institute under a research agreement with the University of North Carolina.

### Cellular effects and cytotoxicity

Enhancing effects and cytotoxicity were examined using modifications of previously described techniques. Briefly, the analogs were tested for their ability to enhance the effectiveness of a splice switching oligonucleotide (SSO623) in HeLa Luc 705 cells that are stably transfected with a luciferase reporter that is responsive to this SSO. The SSO was incubated with cells overnight in complete medium in 24-well plates and then removed prior to addition of test compounds. Cells were incubated with test compounds for 2 h followed by removal of the compound and replacement with fresh complete medium. Cells were incubated for a further 4 h and then harvested for luciferase and protein assay as previously described (22,27). In some cases, times of incubation were altered, as specified in the figure legends. Cytotoxicity was evaluated by treating cells with test compounds under the same conditions as the luciferase assay. The compounds were removed and the cells further incubated for 24 h. Cytotoxicity was determined using the Alamar Blue assay as described (22,27).

### Antisense assays

Enhancing effects of OECs on antisense oligonucleotides were examined using a previously described method (22). Briefly, NIH-3T3-MDR1 cells were incubated overnight with 100 nM anti-MDR1 gapmer oligonucleotide in DMEM medium plus 10% FBS. After the oligonucleotides were removed by rinsing, cells were incubated with test compounds (10 μM) for 2 h. The compounds were removed and incubation continued for 48 h. Cell surface expression of P-glycoprotein (Pgp), the protein product of the *MDR1* gene, was measured using a PE conjugated anti-Pgp antibody (BD Bioscience) and flow cytometry.

### Confocal microscopy

The effect of selected analogs on the subcellular localization of oligonucleotide was examined as follows. Cells were incubated in complete medium with various concentrations of a TAMRA-labeled version of SSO623 and then rinsed and returned to fresh complete medium. Analogs were added and the cells incubated for various periods. Live cells were examined using a confocal microscope with an environmental stage that provided control of temperature and CO_2_. The organization of intracellular organelles was studied using CellLights 2.0 baculovirus vectors that express GFP chimeras of proteins known to associate with particular endomembrane compartments. In some cases the location of the nucleus was delineated by treating the cells with Hoechst 33342 in PBS. A Zeiss LSM710 confocal microscope with 40x oil immersion lens was used to collect images that were then analyzed using the Fiji version of NIH Image J software.

### Lysotracker analysis

HeLa Luc705 cells in complete medium in 24 well plates were treated with various concentrations of selected analogs for 1.5 h. Lyotracker Red^®^ was added to 200 nM and incubated for 15–20 min. Cells were rinsed in 3× in PBS, lysed in 0.2% TX100, and the plates were centrifuged at 4000 rpm. Aliquots of supernatant (100 μl) were distributed to 96 well black plates and Lysotracker fluorescence was measured using a FLUOstar Omega 96 well plate reader. A similar assay of mitochondrial membrane potential using the dye TMRE was performed with a kit as per the manufacturer's instructions.

### Cell death assays

The role of caspases and of apoptosis in the cytotoxicity of OECs was evaluated by incubating cells with the OEC in the presence or absence of the pan-caspase inhibitor QVD-O-Ph. Similarly, the role of lysosomal proteases was examined by incubating OEC–treated cells with or without the protease inhibitor E64d. Plasma membrane permeability was evaluated using a LIVE/DEAD^®^ kit by testing the ability of cells to retain accumulated calcein or to take up ethidium dimer during or after exposure to various concentrations of OEC.

## RESULTS

### Structure activity relationships (SAR) of the analogs

**Figure 1.**
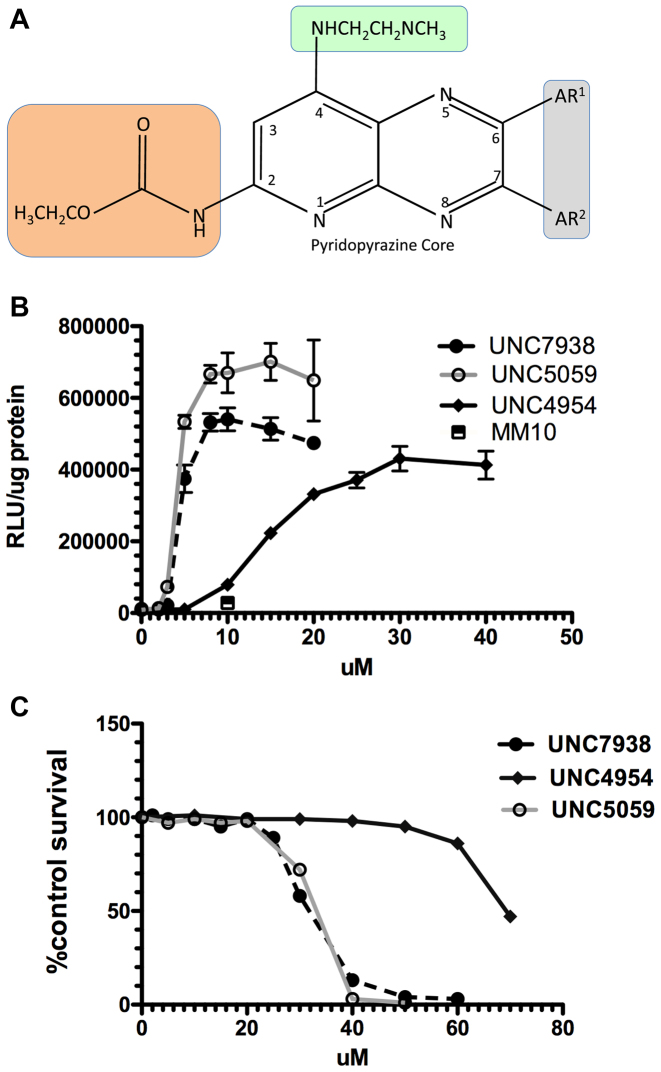
(**A**) Structure of UNC7938 Analogs. The structure of UNC7938 and the various groups that were modified in the analogs synthesized in this study are depicted. AR^1^ and AR^2^ are aromatic groups (gray shaded area). The orange shaded area is the carbonate group. The green shaded area is the tertiary amine group. Detailed structures of analogs are shown in Supplementary Tables S1–S4. (**B**). *OECs Enhance Oligonucleotide Effects*. HeLa Luc 705 cells were incubated in 24-well plates (50,000 per well) with 100 nM SSO623 or its mismatched control (MM) for 16 h in DMEM + 10% FBS, rinsed and then treated with various concentrations of OEC compounds for 2 h. The cells were then rinsed and incubation continued for an additional 4 h in DMEM + 10% FBS. Cells were rinsed twice in PBS and luciferase activity (RLU) and cell protein determined. The square symbol indicates the MM oligonucleotide with UNC7938. Means ± SE. *N* = 3. (**C**) *Cytotoxicity of OECs*. HeLa Luc705 cells were exposed to compounds as in (B) then incubated for 24 h in DMEM + 10% FBS and tested using the Alamar Blue cytotoxicity assay. Means ± SE. *N* = 3.

The parental OEC for this study was the initial hit from our high throughput screen, UNC7938, whose properties have been previously described (22). The structure of UNC7938 is depicted in Figure 1A with the groups that were modified in this study indicated by highlighting. Various analogs of UNC7938 were designed, synthesized, and tested for their effectiveness in enhancing the ability of a SSO to induce expression of a luciferase reporter gene in a cell-based assay. They were also examined for cytotoxicity under the same conditions used for the luciferase assay. A typical luciferase and cytotoxicity experiment is depicted in Figure 1B and C comparing UNC7938 to two new analogs UNC4954 and UNC5059. In UNC4954 the carbamate of UNC7938 is converted to a free amine, whereas in UNC5059 the dimethyl amine of UNC7938 is replaced with a pyrrolidine. Based on experiments of this type two key parameters were measured for the various analogs: (i) the concentration of compound needed for 50% of the maximal enhancement of SSO activity (EC50); (ii) the concentration of compound that caused 50% cytotoxicity as compared to untreated control (TC50). The ratio of TC50/EC50 provides a useful measure of toxicity versus potency in the cell culture setting. In these studies, any compound that failed to produce at least a five-fold enhancement of SSO activity upon treatment with 30 μM compound was deemed to be inactive and not studied further. Table 1 provides a list of the compounds tested and their EC50 and TC50 values.

**Table 1.** EC50 and TC50 summary for analogs

Compound	EC50 (μM)	TC50 (μM)	TC50/EC50 ratio
UNC7938	3.6	28.0	7.7
UNC4954	14.0	55.0	3.9
UNC5059	4.3	33.0	7.6
UNC5103	4.0	23.0	5.8
UNC5127	21.0	47.5	2.3
UNC5163	3.8	21.5	5.7
UNC4093	>30	ND	-
UNC4102	>30	ND	-
B-36	>30	ND	-
B-48	8.0	80.0	10.0
B-65	14.0	80.0	5.7
B-78	>30	ND	-
B-116	4.5	41.0	9.1
B-128	8.5	41.0	4.8
B-136	4.0	20.0	5.0
B-141	1.8	13.0	7.2
B-152	3.1	18	5.8
B1–225	>30	ND	-
B1–250	>30	ND	-
B1–257-2	>30	ND	-
B1–252-P	20	54.0	2.7

ND = not determined.

EC50 and TC50 values are averages from two to three experiments where each experiment used the same batch of cells and stock of compound for the functional assay and the cytotoxicity assay.

The detailed structures of UNC7938 and its analogs, as well as a recapitulation of their TC50/EC50 ratios, are depicted in Supplementary Tables S1–S4 of Supplementary Information I. The synthesis of compounds produced at UNC is fully described in Supplementary Information II while synthesis of compounds from Chemogenics Biopharma is described in detail in Supplementary Information III. The OEC analogs prepared in this study focused on exploring three aspects of the structure of UNC7938: (i) the phenyl rings; (ii) the tertiary amine and its alkyl linker; (iii) the carbamate group.

Our studies of EC50 and TC50 values revealed several important structure–activity relationships. First, the aromatic rings are required for activity. As shown in Supplementary Table S1, removal of one ring, as in compounds UNC4102 and UNC4093, rendered the compounds inactive. Further, substitution of the rings with four to eight carbon alkyl chains did not support activity, as demonstrated by compounds B1–225, B1–250 and B1–257-2. An analog in which the aromatic rings were replaced by cyclohexyl rings, B-252-P, displayed some activity but was not as potent or as efficacious as UNC7938. Thus both phenyl rings of the pyridopyrazine core appear to be playing a key role and may be involved in interactions with components of endosomal membranes, the presumed site of action of these compounds.

Second, the tertiary amine moiety was also varied to evaluate effects on potency and toxicity. As seen in Supplementary Table S2, replacement of the dimethyl amine with a larger pyrrolidine (UNC5059) or a smaller primary amine (UNC5163) resulted in only minor effects on toxicity or potency. Similarly elongation of the ethyl chain, which connects the dimethyl amine to the pyridopyrazine core, to a propyl chain (UNC5103) had minimal effects. However, replacement of the tertiary amine with a morpholino group, as in UNC5127, resulted in a loss of potency and a reduction in the TC50/EC50 ratio. The pKa of the morpholine group is substantially lower than that of the dimethyl amine; this may affect the behavior of the compound in the endomembrane environment.

Third, the carbamate group of UNC7938 was varied. This group is not essential for activity since compound UNC4954, in which the carbamate is simplified to a primary amine, retains activity albeit about four fold less than UNC7938 (see Supplementary Table S1). This region of the molecule was further explored by replacing the carbamate group with more stable urea derivatives containing various modifications (see Supplementary Table S3). This provided a series of molecules with widely differing properties. Concentration response curves for several of these urea-containing compounds are presented in Supplementary Figure S1a and b. While compound B-48 with a piperidine moiety was quite active and relatively non-toxic, replacement with a morpholine (B-36) led to complete loss of activity. The addition of a CF3 group to the piperidine of B-48 (B-116) resulted in a derivative that was more potent as well as somewhat more toxic than B-48, while the replacement of the CF3 with gem-dimethyls as in B-128 did not have a substantial effect on the potency. Both B-48 (TC50/EC50 = 10.0) and B-116 (TC50/EC50 = 9.1) demonstrate a toxicity/potency ratio that is improved over that of UNC7938 (TC50/EC50 = 7.7).

Additional analogs were made that rigidified the flexible tertiary amine side chain (see Supplementary Table S4). Based on prior results these compounds were prepared in the context of various urea groups at the carbamate position. Overall, the piperazine-containing compounds B-136, B-141 and B-152 were potent but also quite toxic resulting in TC50/EC50 ratios lower than UNC7938 (Supplementary Figure S1c).

In addition to testing effects with SSOs in HeLa human tumor cells, we also examined the influence of several OECs on the action of an antisense gapmer in multi-drug resistant NIH-3T3 mouse fibroblasts. As seen in Supplementary Figure S2, the OECs UNC7938, UNC5103 and B-116 each substantially enhanced the ability of an anti-MDR1 gapmer to reduce expression of P-glycoprotein, the *MDR1* gene product. Thus, the OECs are effective in quite different cell types and using different forms of oligonucleotide.

The SAR described above demonstrates the key roles of the dual phenyl groups as well as the basic amine of UNC7938, while modifications to the carbamate can also substantially influence the potency and toxicity of the OEC. In the series of compounds generated thus far we have not identified any that are active below one micromolar. Additionally, we have not yet identified molecules that display a major advantage over the parental compound UNC7938 in terms of the ratio of potency to toxicity. However, only a limited number of compounds have been evaluated to date and additional modifications and exploration of the carbamate group and the pyridopyrazine core are likely to lead to further improvement in the therapeutic ratio.

Importantly, availability of a set of analogs of varying potency and toxicity facilitated investigation of basic aspects of the actions of OECs. Thus, in the sections below we evaluate whether the new analogs function similarly to UNC7938. We also more precisely define the intracellular locus of action of these compounds. Finally, we explore their mechanism of toxicity.

### The effect of analogs on the intracellular distribution of oligonucleotides is similar to that of the parent compound

**Figure 2.**
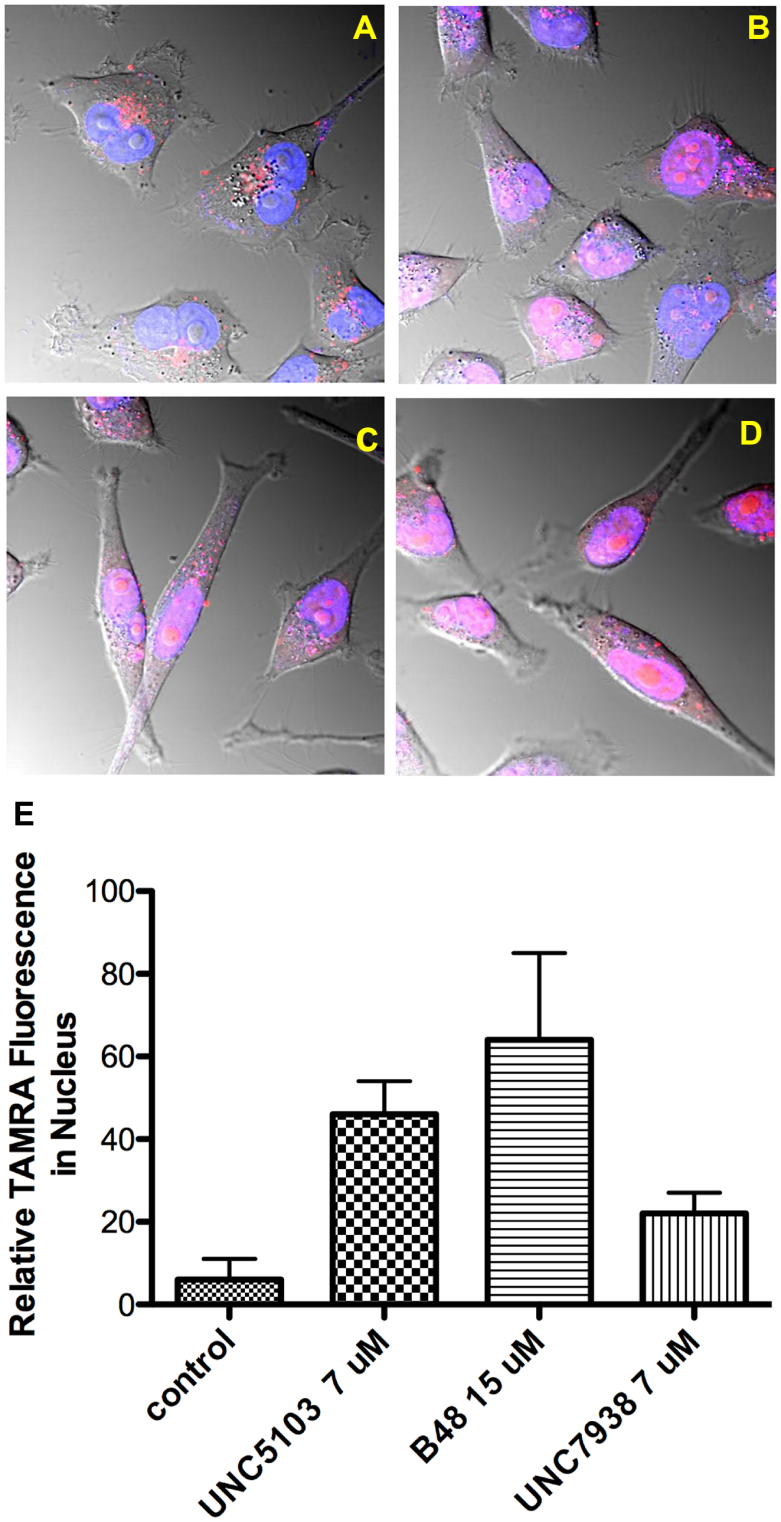
OEC Effects on Re-localization of Oligonucleotide to the Nucleus. HeLa Luc 705 cells (50,000) were seeded into glass-bottom dishes and incubated at 37°C for attachment. TAMRA labeled SSO 623 (2.5 μM) was added into medium and the cells incubated for 24 h. Cells were washed with PBS and then placed back in medium and treated with OECs for 2 h or maintained as controls. Cells were rinsed after drug treatment. Live cells were imaged using a confocal microscope. (**A**), control cells; (**B**), cells treated with 15 μM B-48; (**C**), 7 μM UNC7938; (**D**), 7 μM UNC5103. Images are composites of TAMRA (red), Hoechst (blue) and DIC images and the pink coloration in indicates overlap of the TAMRA and Hoechst fluorescence. (**E**) Quantitates the TAMRA fluorescence in the nucleus for controls versus treated cells, as measured using Fiji software, with Hoechst stain used to delineate the nucleus. The differences between treated and control cells were significant at the 0.5% level for all compounds evaluated using the paired *t*-test.

**Figure 3.**
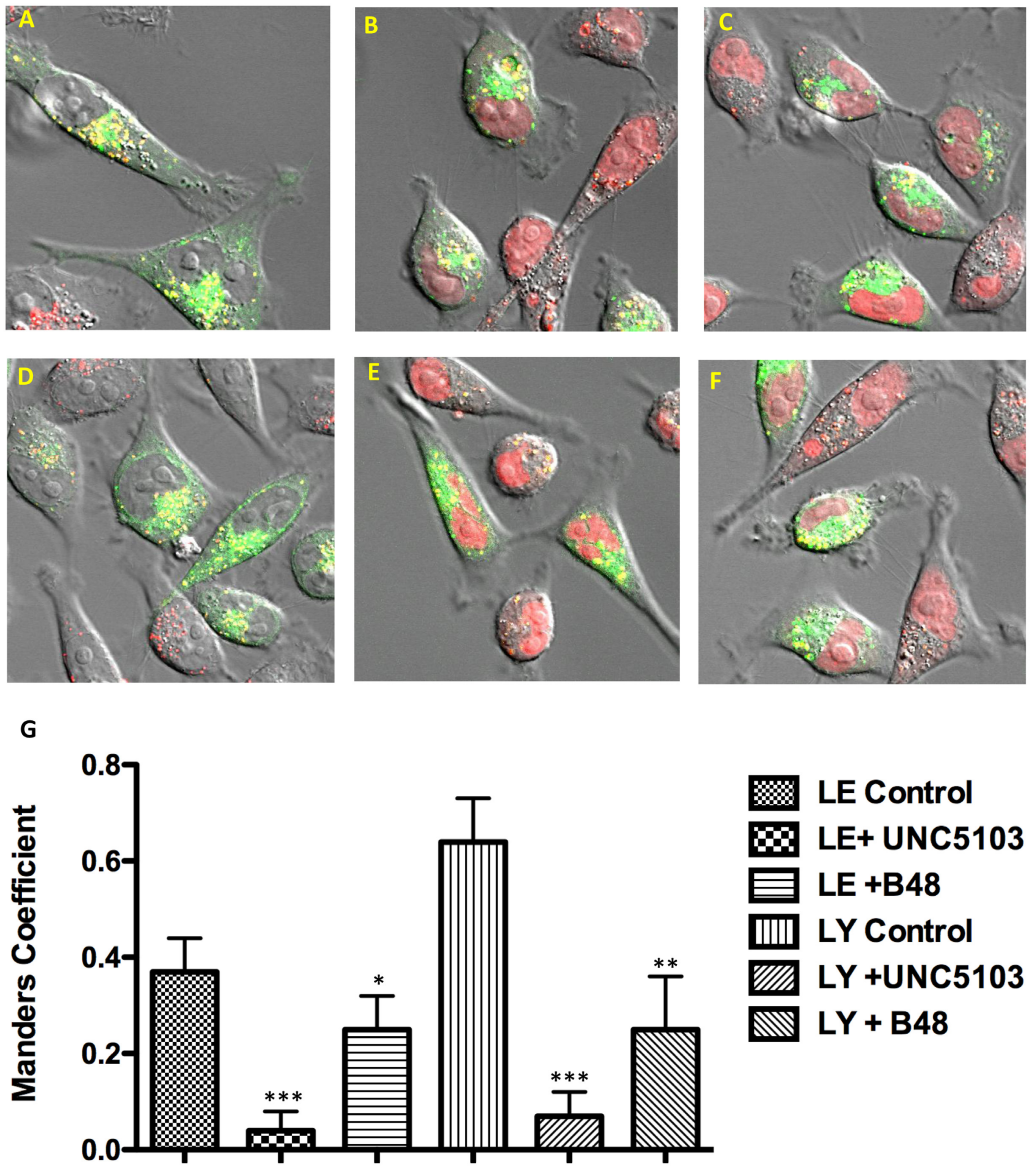
Effects of OECs on Endomembrane Compartments. HeLa Luc 705 cells were dually labeled with TAMRA SSO and, by using baculovirus vectors, with GFP-Rab7 or GFP-LAMP1 as markers for late endosomes (LE) or lysosomes (LY) respectively. Subsequently cells were treated for 90 min with OECs or maintained as controls. Composite images showing TAMRA fluorescence (red), GFP (green) and DIC are provided. Overlap is indicated in yellow. (**A**) LY control; (**D**) LE control; (**B**, **E**) treated with 15 μM B-48; (**C**, **F**) treated with 9 μM UNC5103. Note the ‘empty’ nuclei in controls versus the partial redistribution of TAMRA SSO to the nucleus in treated cells. The co-localization of red and green fluorescence is quantitated in (**G**) as the Manders Coefficient: significance at the 0.5% level (***), 1.0% level (**), 2.5% level (*).

Using the available compounds we investigated the cellular mechanisms of action and toxicity of OECs. One aspect involved using confocal microscopy to evaluate effects on the subcellular distribution of oligonucleotides. Based on previous studies with UNC7938 (22), we anticipated that the active analogs (OECs) would work in a similar manner and cause release of oligonucleotides from endomembrane compartments. This has been confirmed. For example, as illustrated in Figure 2A confocal microscopy images show that in control cells a TAMRA-labeled fluorescent oligonucleotide was primarily found in cytosolic vesicles. However upon treatment with B-48, one of the active analogs, there was a substantial re-localization of the fluorescent oligonucleotide to the nucleus (Figure 2B). Similar re-localization was also observed with UNC7938 itself and with UNC5103 and UNC4954, other active analogs (Figure 2C, D and Supplementary Figure S3). The extent of redistribution to the nucleus is quantitated in Figure 2E and is clearly quite substantial. In these studies, as well as those shown in Figure 3, a high concentration of fluorescent oligonucleotide was used to allow visualization of the material. Additionally relatively high concentrations of the OECs were used in order to obtain clear-cut effects.

We also examined the effects of various analogs on the co-localization of a fluorescent oligonucleotide with specific endomembrane compartments through use of baculovirus vectors that express GFP chimeras of marker proteins for those compartments (Figure 3). As seen in Figure 3A, in control cells there was substantial overlap of a TAMRA oligonucleotide with GFP-LAMP1, a marker for lysosomes (LY). Upon treatment with UNC5103 or with B-48 there was reduced co-localization with lysosomes and increased distribution of the oligonucleotide to the nucleus (Figure 3B and C). Similarly, using GFP-Rab7 as a marker for late endosomes (LE), compounds UNC5103 or B-48 also induced changes in co-localization of the GFP and TAMRA labels as well as increased nuclear accumulation (Figure 3D–F). The degree of co-localization was quantitated using the Manders Correlation Coefficient as depicted in Figure 3**G**. Interestingly, despite the fact that both compounds clearly caused an increase in nuclear localization of oligonucleotide, there were variations between UNC5103 and B-48 in terms of endomembrane co-localization. Thus the difference in the Manders parameter between control cells and UNC5103 treated cells was highly statistically significant for the LE and LY markers. However, the effect of B-48 was less statistically significant for both markers. The concentrations of each compound used here were chosen on the basis of approximately equal effects in the luciferase induction assay. These variations may reflect subtle differences among the compounds, but may also reflect the fact that it is difficult to establish clear-cut quantitative relationships between functional assays and confocal observations of oligonucleotide intracellular distribution. Nonetheless, the results clearly show that treatment with OECs results in redistribution of oligonucleotide from endomembrane compartments to the cytosol and nucleus. Thus the new compounds act in a manner similar to UNC7938, the parental compound.

Although the OECs clearly alter the permeability of endomembranes, they do not have substantial effects on the overall organization of major endomembrane compartments. This is illustrated in Supplementary Figure S4. Here, we again used baculovirus vectors to express GFP chimeras of marker proteins that are associated with late endosomes, lysosomes, or the Golgi apparatus. The appearance of these organelles was then visualized by confocal microscopy in cells treated with OECs or maintained as controls. We did not observe any major differences in the intensity or distribution of organelle labeling. This is particularly clear for the Golgi that has a very well demarcated perinuclear distribution. Although these qualitative observations cannot rule out subtle changes in organelles, they suggest that the effect of the OECs is not due to an overall disruption of organelle structures but rather to changes in their permeability.

### Subcellular mechanism of OEC action

The OECs discovered thus far have potencies only in the micromolar range, making the identification of their molecular target(s) via typical proteomic or lipidomic techniques very challenging. However, we have sought to more precisely define their action at the subcellular level. Although microscopic observations are important, the overall subcellular distribution of oligonucleotide may or may not reflect its functional behavior. Some studies have suggested that there may be productive and non-productive pathways of oligonucleotide trafficking; however, these may be difficult to distinguish morphologically. Thus we sought to better define the cellular site of action of the OECs using functional assays.

**Figure 4.**
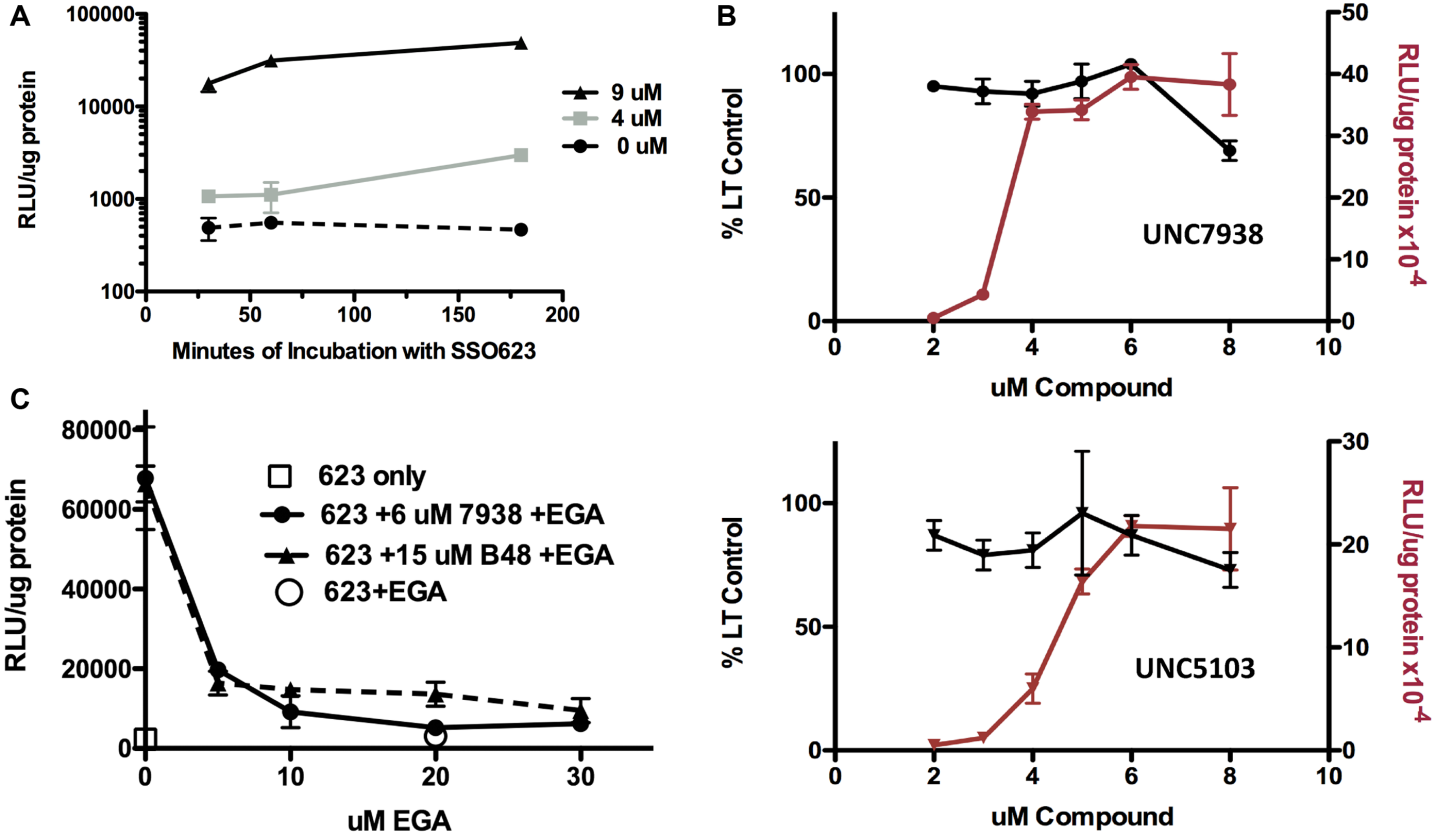
Subcellular Mechanism. Several approaches were used to more explicitly delineate the subcellular locus of action of the OECs. (**A**). *Kinetics of the OEC Effect*. Cells were incubated with 500 nM SSO623 for various periods, rinsed and then treated with 0, 4 or 9 μM UNC7938 for 30 min. After an additional two rinses the cells were incubated in medium for 3 h to allow luciferase expression. Means and SE. *N* = 3. (**B**) *Comparison of OEC Effects on Lysotracker Accumulation and Luciferase Induction*. In parallel experiments using the same stocks of compounds, dose-effect curves were established for inhibition of Lysotracker accumulation (black lines) and for induction of luciferase (red lines). A narrow concentration range was chosen so as to be able to examine the detailed profiles. The incubation conditions for the SSO and for the OECs were the same as for Figure 1. Lysotracker Red was used at 200 nM. Means and SE. *N* = 3. (**C**) *EGA Inhibition of the Effect of OECs*. Cells were pre-incubated with various concentrations of EGA for 15 min. The cells were then treated with 500 nM SSO623 in the presence of EGA for 1 h. Cells were rinsed and then treated with 6 μM UNC7938 or 15 μM B-48 in the presence of EGA for 1 h and then rinsed 2× in medium. Cells were then incubated for 3 h in medium followed by processing for luciferase induction. Means ± SE. *N* = 3.

One approach was to examine the kinetics of the process. With unassisted or ‘gymnotic’ delivery of oligonucleotides there is usually a long lead-time between the initial uptake process and the manifestation of functional changes (28,29). In contrast, however, when cells were treated with UNC7938 there was a strong enhancing effect after as little as 30 min incubation with oligonucleotide (Figure 4A). This suggested that the OEC may affect oligonucleotide release at a relatively early step in the intracellular trafficking process.

To further pursue this we made use of the fact that there is a pH differential between early endomembrane compartments and late stage compartments such as lysosomes (16). Lysotracker^®^ dyes are trapped in low pH compartments, particularly lysosomes, and their overall accumulation in cells reflects this trapping process (30). An increase in the permeability of the lysosome membrane will perturb the pH gradient between the cytosol and the interior of the lysosome resulting in reduced Lysotracker accumulation. Thus we used the Lysotracker Red^®^ probe to explore the relationship between OEC action and the permeability of low pH compartments. Cells were incubated with various concentrations of the OECs UNC7938 or UNC5103 and then tested in parallel experiments for luciferase induction or Lysotracker Red accumulation. As seen in Figure 4B, OEC concentrations that were relatively low but still quite effective for enhancement of luciferase induction had little influence on Lysotracker Red accumulation, whereas higher concentrations did so. Thus increasing the concentrations of UNC7938 or UNC5103 from 2 to 6 μM resulted in dramatic increases in luciferase induction with little effect on Lysotracker levels. This suggests that pharmacologically significant release of oligonucleotides can take place from pre-lysosomal endomembrane compartments that have a higher pH and thus do not accumulate Lysotracker dye to the same degree as lysosomes. However, at high OEC concentrations oligonucleotide release from lysosomes may also occur.

To further define the site of action of the OECs we made use of a recently discovered small molecule that has the unique effect of inhibiting subcellular trafficking at the early endosome stage. EGA has been shown to block the transfer of certain toxins and viruses from early endosomes to downstream acidified compartments (31). In our experiments, we incubated cells with SSO623 in the presence or absence of EGA with the intent of slowing the progression of the oligonucleotide from early to late endomembrane compartments. As shown in Figure 4C, micromolar concentrations of EGA almost completely ablated the enhancing effect of OECs UNC7938 or B-48 without any evidence of cytotoxicity. In agreement with the original publication (31) we found that EGA had only modest effects on cellular uptake of oligonucleotide and on intracellular pH (see Supplementary Figure S5) confirming that EGA acts primarily by retarding oligonucleotide trafficking between early endosomes and downstream compartments. Confocal microscopy of the subcellular distribution of a fluorescent oligonucleotide also indicated that EGA retards trafficking of internalized material to downstream compartments (Supplementary Figure S6). Since the OECs failed to enhance luciferase induction in the presence of EGA, this indicates that the OECs cannot release oligonucleotide entrapped in early endosomes. In summary, the observations in Figure 4B and C strongly suggest that OECs release oligonucleotides from an endomembrane compartment that is downstream of early endosomes but upstream of strongly acidic compartments.

### Effects of OECs on other intracellular membranes

**Figure 5.**
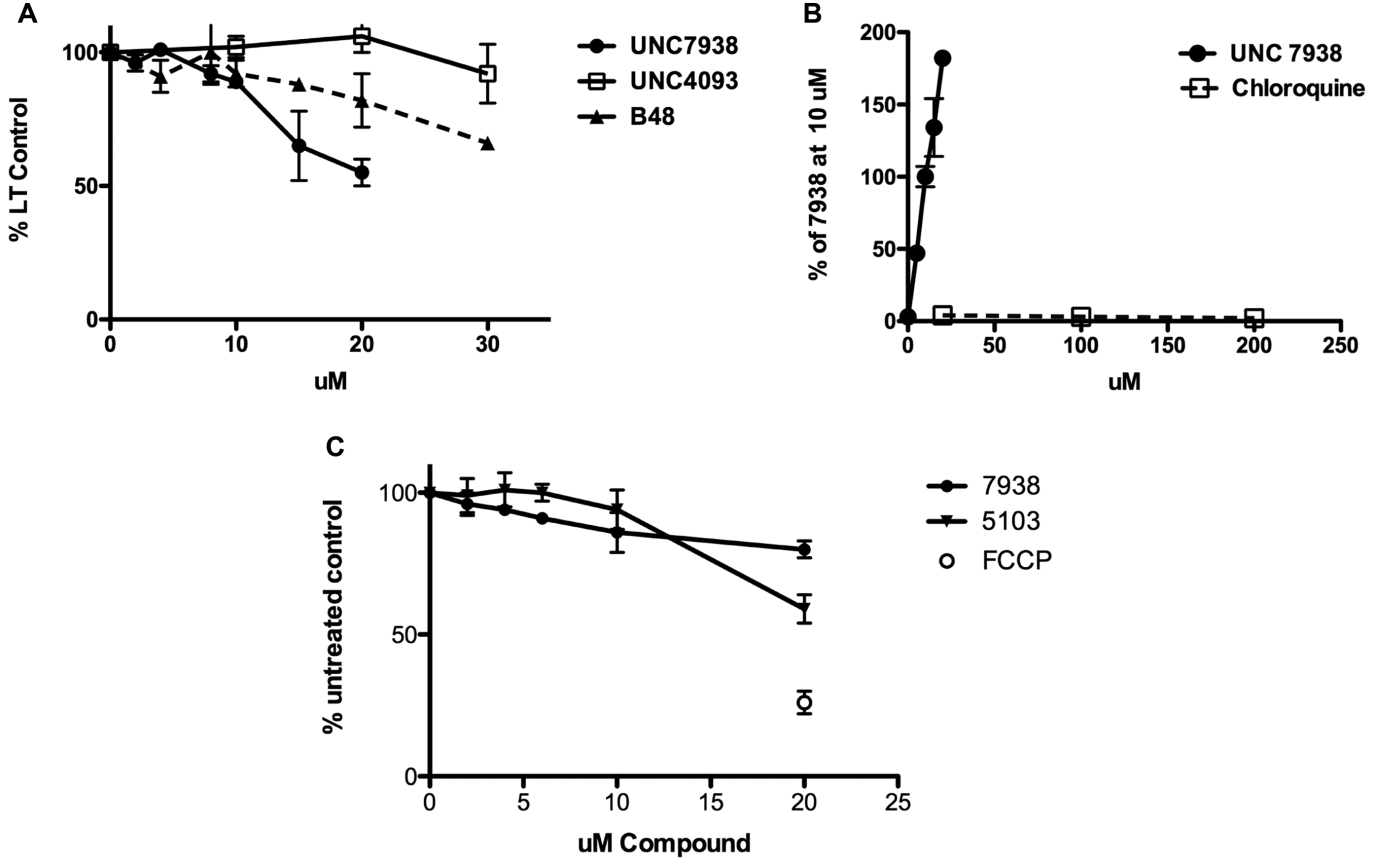
OEC Effects on Other Intracellular Membranes. (**A**). *Lysotracker Uptake*. Accumulation of Lysotracker Red in HeLa Luc 705 cells was measured after a 1.5 h exposure of cells to the indicated concentrations of various analogs. Means ± SE. *N* = 3. (**B**) *UNC7938 vs Chloroquine*. HeLa Luc705 cells were exposed to SSO623 and then treated with various concentrations of UNC7938 or chloroquine for 2 h and then tested for luciferase induction following the methods described in Figure 1. Means ± SE. *N* = 3. (**C**) *OEC Effects on Mitochondrial Membrane Potential.* Cells were incubated with various concentrations of UNC7938 or UNC5103 for 80 min. At this point TMRE was added to 500 nM and the incubation continued for 20 min. Cells were then rinsed in PBS, lysed in 0.2% TX100, and the accumulation of TMRE in the lysate was quantitated using a 96 well plate reader. FCCP is known to reduce the mitochondrial membrane potential and was used as a positive control. Means ± SE. *N* = 3.

Although OECs primarily affect an intermediate endosomal compartment, we also evaluated the effects of these compounds on lysosomes and other organelles by using chemical probes that can monitor the status of particular membrane-bound compartments. As mentioned above, the Lysotracker Red^®^ probe can be used to explore lysosome permeability. As seen in Figure 5A, various OEC analogs affected Lysotracker Red accumulation at different concentrations. For example, UNC7938, a highly active OEC, reduced Lysotracker accumulation substantially, whereas UNC4093, a compound that is inactive in the luciferase induction assay, had little effect on Lysotracker accumulation over the concentration range tested. The compound B-48, which is quite active but less toxic than UNC7938, displayed an intermediate profile. Thus when used at concentrations higher than those needed to enhance luciferase induction, the active OECs do affect lysosome permeability. However, UNC7938 and its analogs are clearly not typical lysosomotropic compounds. This is illustrated in Figure 5B. Whereas UNC7938 strongly enhanced the effect of a SSO on the luciferase reporter, chloroquine failed to do so even at very high concentrations. This suggests that release of oligonucleotides from lysosomes does not strongly contribute to the functional effect. Other investigators have also found that chloroquine does not enhance effects of single stranded phosphorothioate oligonucleotides (32). Thus, the effects of UNC7938 and its analogs are distinct from those of typical lysosomotropes such as chloroquine that act by pH driven accumulation in highly acidic endomembrane compartments followed by osmotic lysis (33).

We also examined effects of our compounds on mitochondria, another membrane bound subcellular organelle. This was done using TMRE, a probe that monitors mitochondrial membrane potential and is thus very sensitive to changes in membrane permeability. As seen in Figure 5C concentrations of UNC7938 or UNC5103 that were very effective in enhancing luciferase induction had little effect on mitochondrial membrane potential. Thus, when used at low but effective concentrations, the OECs display selectivity toward intermediate components of the endomembrane trafficking system rather than causing a general perturbation of intracellular membranes.

### Mechanisms of OEC cytotoxicity

The study of cell death has become an active and complex field. In addition to the well established mechanisms of apoptosis and necrosis, recent work has described multiple additional death pathways including necroptosis, pyroptosis, autophagic cell death and lysosome-mediated cell death (34–38). Here, we have used several simple but informative assays (39) to provide an initial delineation of the cell death process that is triggered by exposure to high concentrations of OECs.

**Figure 6.**
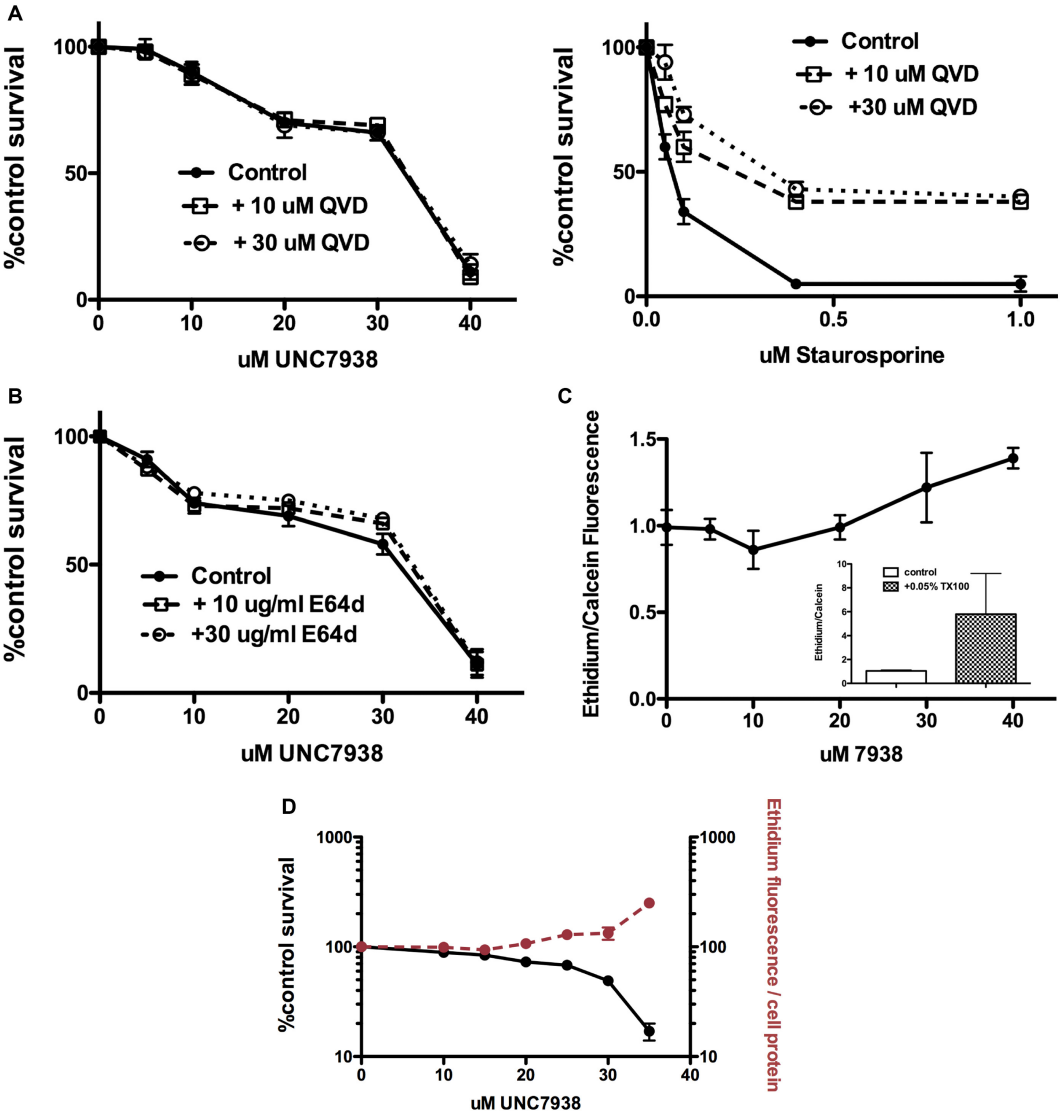
Mechanism of Cytotoxicity. (**A**) Lack of a Role for Caspases. Cells (75,000/well) were treated with UNC7938 or staurosporine for 2 h, both in the presence of 10 or 30 μM QVD a pan–caspase inhibitor. After removal of the test compounds incubation was continued overnight in the presence of QVD. Thereafter the Alamar Blue cytotoxicity assay was performed. Means ± SE. *N* = 3. Left panel: UNC7938. Right panel: staurosporine. (**B**) *Lack of Effect of a Lysosomal Protease Inhibitor*. Cells (75,000/well) were treated with UNC7938 for 2 h in the presence of 10 or 30 μg/ml E64d, a cathepsin inhibitor. After removal of the test compound incubation was continued overnight in the presence of E64d. Thereafter the Alamar Blue cytotoxicity assay was performed. Means ± SE. *N* = 3. (**C**) *OEC Effects on Plasma Membrane Permeability*. Cells were incubated with various concentrations of UNC7938 for 2h in medium, rinsed in PBS, and then incubated with 4 μM ethidium dimer and 2 μM Calcein AM in PBS for 30 min. Cells were rinsed twice in PBS and then lysed in 0.3 ml of 0.2% TX100 in PBS. Ethidium and calcein fluorescence was measured using the appropriate settings on a plate reader. The ethidium/calcein ratio, normalized to untreated controls, is shown. Means ± SE. *N* = 6. The inset depicts cells permeabilized with the detergent TX100 as a positive control. (**D**) *OEC Effects on Plasma Membrane Permeability Continued*. Cells were co-incubated in medium with 4 μM ethidium dimer and various concentrations of UNC7938 for 2 h. Cells were rinsed twice in PBS and then lysed in 0.3 ml of 0.2% TX100 in PBS. Ethidium fluorescence was measured using a plate reader. The ethidium/cell protein ratio, normalized to untreated controls, is shown. Cytotoxicity measured using the Alamar Blue assay is also shown. Means ± SE. *N* = 3.

Caspases play a central role in both the mitochondrial and extrinsic pathways of apoptosis (40), thus it is important to evaluate whether they contribute to OEC cytotoxicity. To do this we made use of the well-known cell permeable pan-caspase inhibitor QVD-O-Ph (41,42). As seen in Figure 6A (left panel) QVD-O-Ph had essentially no effect on the cytotoxicity profile of UNC7938; however, the same concentrations of QVD-O-Ph dramatically attenuated the toxicity of staurosporine, a typical apoptosis inducer (Figure 6A, right panel). Thus OEC-mediated cell death is caspase-independent and does not involve a typical apoptotic process.

Since OECs at higher concentrations clearly affect the permeability properties of the lysosome, we also examined the possibility that escape of lysosomal hydrolases might contribute to cytotoxicity. We utilized E64d a cell permeable cysteine protease inhibitor that is known to act on several lysosomal cathepsins. Cells were treated with UNC7938 with or without exposure to concentrations of E64d that have been shown to strongly inhibit intracellular cathepsins in other systems (43,44). As seen in Figure 6B, E64d had only minimal effects on the cytotoxicity profile of UNC7938. This suggests that lysosomal cathepsins are not major contributors to OEC cytotoxicity. A caveat is that lysosomes contain many different hydrolases including ones not affected by E64d. However, several studies of lysosome-mediated cell death have shown that cathepsin inhibitors usually substantially affect this process (45,46).

An increase in plasma membrane permeability is a hallmark characteristic of necrosis. We assessed possible OEC effects on plasma membrane permeability by use of a Live Dead® kit. Cells are incubated with the membrane permeable dye Calcein AM that is enzymatically converted to non-permeant calcein and trapped within live cells. Co-incubation with the DNA binding dye ethidium dimer, which does not penetrate live cells, provides a second measure of membrane permeability. Thus the ethidium/calcein fluorescence ratio provides a convenient index of cell membrane integrity. As seen in Figure 6C, in cells first treated with UNC7938 and subsequently incubated with the two dyes the ethidium/calcein ratio was essentially constant up to 20 μM. However, in the 20–40 μM range the ratio significantly increased. In a parallel experiment we co-incubated ethidium dimer with UNC7938 and measured ethidium accumulation in cells (Figure 6D). There was clearly a sharp increase in ethidium uptake in the 20–40 μM range. It is in this concentration range that substantial cytotoxicity is observed for UNC7938 (see Figures 1C and 6D). Similar observations on ethidium uptake versus cytotoxicity were made for compounds UNC5103 and B-116 (see Supplementary Figure S7). Thus, loss of plasma membrane integrity seems to closely parallel cell death. Based on the observations in Figure 6A–D and Supplementary Figure S7, the induction of cytotoxicity by high concentrations of OECs seems to primarily be a necrotic process due to non-selective effects on the plasma membrane.

## DISCUSSION

A wide variety of delivery technologies including many types of lipid and polymer nanoparticles, cell penetrating peptides, and ligand-oligonucleotide conjugates have been used in attempts to improve the pharmacological actions of oligonucleotides (47,48). In some cases these approaches have met with success in the clinic (6). However, there is clearly a need for additional approaches to this difficult problem. Thus, several groups have recently explored the use of small molecules to enhance the effectiveness of oligonucleotides (23,24). In our case we previously used high throughput screening to identify two novel families of oligonucleotide enhancing compounds (OECs) (22,25).

In the current study, we prepared a number of analogs of the original HTS hit compound UNC7938 and evaluated the properties of the analogs. The SAR studies revealed several key aspects of these molecules. First, there is a requirement for two lipophilic six carbon aromatic residues. Neither use of a single aromatic residue nor replacement with two six-carbon aliphatic rings allowed full effectiveness. Second, the tertiary nitrogen plays a key role in activity. Replacement with a morpholino modification caused a substantial reduction in potency and effectiveness. In contrast, restricting the conformation of the tertiary nitrogen led to increased potency, but also increased toxicity. Third, modification at the 2 position of the core structure is tolerated and can lead to a variety of effects. Replacement of the carbamate at position 2 with a piperidine moiety led to a compound (B-48) with good potency and efficacy as well as reduced toxicity thus providing an improved TC50/EC50 ratio. Other modifications at this position led to loss of activity, while still others demonstrated increased potency. Thus this site seems promising for further modifications.

The new analogs seem to work by the same mechanism as the parent compound UNC7938. Thus they release oligonucleotide from endomembrane compartments allowing better access to cytosolic or nuclear target sites. This can be visualized using a fluorescent oligonucleotide in conjunction with markers for the various subcellular compartments. While there are likely subtle differences among the various analogs, the overall mechanism of action seems to be conserved.

In this study, we sought to more precisely define the intracellular locus of action of our compounds. This is challenging since the intracellular trafficking system is extremely complicated and the demarcation of the major endomembrane compartments is often poorly defined. Oligonucleotide internalization leads initially to early endosomes and thence to multi-vesicular bodies (MVBs), late endosomes and lysosomes, with possible minor branches to the Golgi and endoplasmic reticulum (14,49). However, the situation is complex since early endosomes gradually mature into MVBs and thence late endosomes via a process that involves multiple components, including the Rab5 and Rab7 GTPases and their adaptors and effectors, as well as the ESCRT complex, and lipids such as PtdIns(3)P and lysobisphosphatidic acid (16,50,51). Thus there are intermediate stages in this dynamic pathway making it difficult to specify the precise site of action of the OECs. Nonetheless, our data indicate that UNC7938 and its analogs act downstream from early endosomes but upstream of highly acidic compartments. Thus in cells where oligonucleotide is trapped in early endosomes by use of the trafficking inhibitor EGA, there is almost no effect of the OEC. This suggests that the early endosome is refractory to the action of the OECs. When we examined effects of the OECs on oligonucleotide action versus their effects on intracellular pH, we found that strong oligonucleotide effects can be attained at OEC concentrations that do not affect the pH of lysosomes or other highly acidic compartments, thus indicating that the site of OEC action is prior to entry into these compartments. These observations place the locus of action of the OEC at an intermediate stage of the endomembrane trafficking pathway. One possibility might be MVBs since these have a higher pH than late endosomes or lysosomes and are formed relatively early in the trafficking process (50). Interestingly, a recent study used siRNA screening to identify a component of the ESCRT complex, which is involved in MVB formation, as an important factor in the trafficking of oligonucleotides (52). Additionally, two reports using advanced confocal microscopy to study the trafficking of siRNA delivered in lipid nanoparticles concluded that the siRNA is released from a hybrid early-late endosomal compartment (18) or at an intermediate stage between Rab 5 positive early endosomes and Rab7 positive late endosomes (53). A recent study of trafficking of phosphorothioate antisense oligonucleotides suggested that ASO release took place at the late endosome stage (28). Taken together, these studies indicate that an intermediate trafficking stage between early and late endosomes, possibly MVBs, plays a central role in the functional intracellular delivery of oligonucleotides. Thus the OECs described here seem to act at the same key stage of oligonucleotide delivery as takes place with ‘free’ oligonucleotides or with lipid nanoparticles.

In terms of OEC toxicity, there seems to be no role for typical caspase-dependent apoptosis since a pan-caspase inhibitor had no effect on the cytotoxicity profile. A cathepsin inhibitor only slightly affected the cytotoxicity curve suggesting a limited role for lysosomal proteases, despite the fact that lysosome permeability is increased by the OECs. The strongest correlation was between OEC effects on plasma membrane permeability and the onset of cell death, suggesting a process of necrosis (39). However, the exact mechanism of toxicity for the OECs, or indeed for most oligonucleotide delivery methods (54), is currently only partially defined.

In summary, this family of OECs release oligonucleotides from an intermediate compartment in the endomembrane trafficking pathway. This presumably is due to a preferential interaction between the OEC and a lipid or protein component that is enriched in the limiting membrane of the target compartment. However, at higher concentrations the OECs affect lysosomal membranes and finally plasma membranes, thus leading to cell death. The analogs developed thus far have allowed identification of key groups on the parent molecule UNC7938. However, they have not yielded compounds that demonstrate a major improvement in selectivity. A key for future development of this type of OEC will be to increase the selectivity for the target compartment versus other cellular membranes thus leading to an improved TC50/EC50 ratio. In addition to further work on the current family of OECs, our identification of the target compartment for oligonucleotide release will hopefully encourage further studies on small molecule enhancers. This might be approached either through phenotypic screening with additional compound libraries, or by designing small molecules to affect key proteins that are enriched in endosomal membranes. Hopefully, such studies will lead to increased selectivity that will permit expanded use of OECs in the *in vivo* context.

## Supplementary Material

Supplementary Data
