# Supplementary material for: Structure–activity relationships and cellular mechanism of action of small molecules that enhance the delivery of oligonucleotides
Source: Nucleic Acids Res. 2018 Jan 18;46(4):1601–13. doi: 10.1093/nar/gkx1320 (PMC5829638; doi:10.1093/nar/gkx1320)

**SUPPLEMENTARY INFORMATION I**

**Supplementary Tables 1-4: Structure Activity Relationships**

**Table S1:**

| **Compd** | **R1** | **R2** | **R3** | **EC50 (uM)** | **TC50 (uM)** | **TC50/EC50** |
| --- | --- | --- | --- | --- | --- | --- |
| UNC  7938 |  |  |  | 3.6 | 28.0 | 7.7 |
| UNC  4093 | **H** |  |  | >30 | NT |  |
| UNC  4102 |  | **Me** |  | >30 | NT |  |
| UNC  4954 |  |  | **H** | 14.0 | 55.0 | 3.9 |
| B1-225 |  |  |  | >30 | NT |  |
| B1-250 |  |  |  | >30 | NT |  |
| B1-257-2 |  |  |  | >30 | NT |  |
| B1-252P |  |  |  | 20.0 | 54.0 | 2.7 |

**Table S2:**

| **Compd** | **R1** | **R2** | **R3** | **EC50uM** | **TC50**  **uM** | **TC50/EC50** |
| --- | --- | --- | --- | --- | --- | --- |
| UNC  5059 |  |  |  | 4.3 | 33.0 | 7.6 |
| UNC  5103 |  |  |  | 4.0 | 23.0 | 5.8 |
| UNC  5127 |  |  |  | 21.0 | 47.5 | 2.3 |
| UNC  5163 |  |  |  | 3.8 | 21.5 | 5.7 |

**Table S3**:

| **Compd** | **R3** | **EC50**  **uM** | **TC50**  **uM** | **TC50/EC50** |
| --- | --- | --- | --- | --- |
| B-48 |  | 8.0 | 80.0 | 10 |
| B-36 |  | >30 | NT |  |
| B-78 |  | >30 | NT |  |
| B-116 |  | 4.5 | 41.0 | 9.1 |
| B-128- |  | 8.5 | 41.0 | 4.8 |
| B-65 |  |  |  |  |

**Table S4:**

| **Compd** | **R2** | **R3** | **EC50**  **uM** | **TC50**  **uM** | **TC50/EC50** |
| --- | --- | --- | --- | --- | --- |
| B-136 |  |  | 4.0 | 20.0 | 5.0 |
| B-141 |  |  | 1.8 | 13.0 | 7.2 |
| B-152 |  |  | 3.1 | 18 | 5.8 |

**Supplementary Figures**

Supplementary Figure 1a,b. *Structure Activity Relationships of Selected Analogs*. Analogs B-36, B-48, B-65 and B-78 have a urea modification in place of the carbamate of UNC7938. The luciferase induction profiles (a) were established using the same conditions as in Figure 1 of the main text. Cytotoxicity profiles (b) of the two active analogs (B-48, B-65) were established using the Alamar Blue assay. Means and standard errors. N=3.


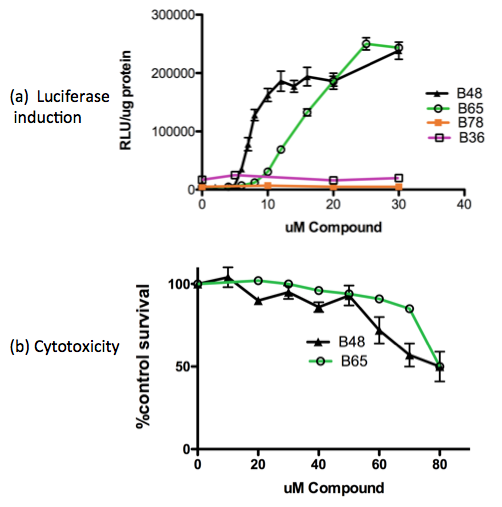


Supplementary Figure 1c. *Comparision of B-141 to UNC7938*. These two compounds were tested for luciferase induction over a narrow range of concentrations. The plot shows that B-141 is more potent (plot left shifted) than UNC7938.


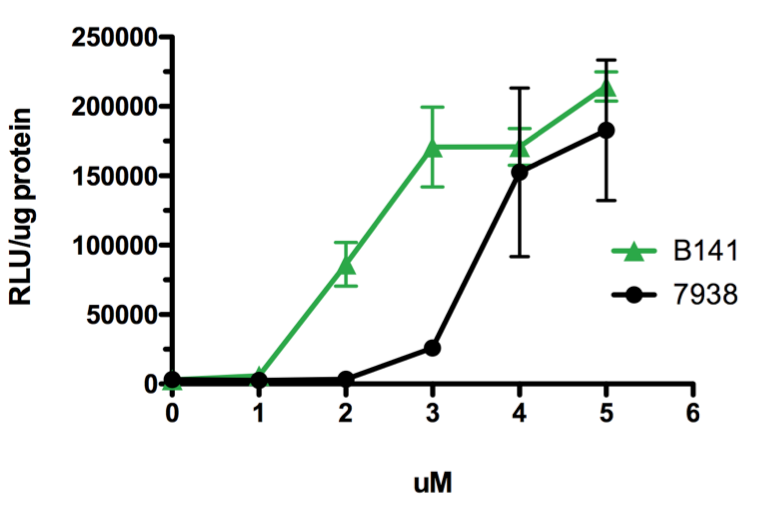


Supplementary Figure 2*. Enhancing Effects on a MDR1 Antisense Oligonucleotide*: NIH-3T3-MDR1 cells were incubated with 100 nM anti-MDR1 antisense oligonucleotide overnight. After removal of the oligonucleotide, the cells were incubated for with OECs UNC7938, UNC5103 and B-116 (10 µM each) for 2 h. After removal of the compounds, the cells were further incubated for 48 h and then assayed for cell surface expression of Pgp using a monoclonal antibody and flow cytometry. Single treatment with the antisense oligonucleotide alone produced a slight decrease of surface expression of Pgp. Further treatment with each OEC dramatically enhanced antisense action, and dramatically reduced Pgp expression as indicated by a left shift of the cytometry profile.


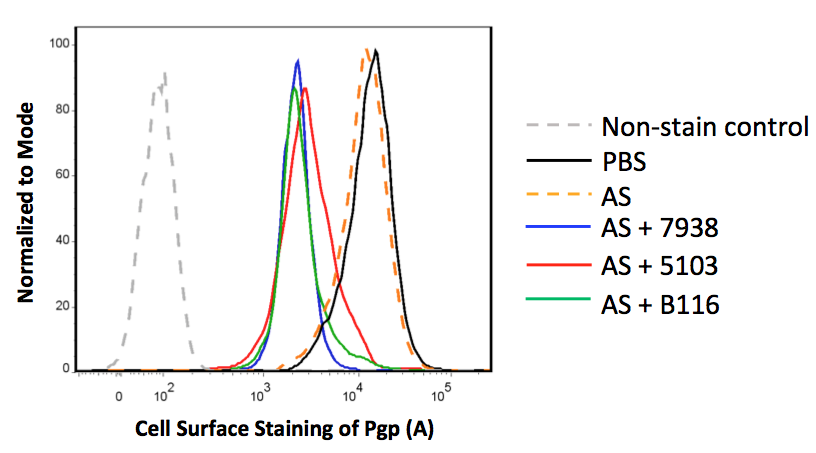


Supplementary Figure 3*. Redistribution of Fluorescent Oligonucleotide to the Nucleus.* HeLa Luc 705 cells were incubated overnight with 2.5 µM TAMRA labeled SSO623 and then rinsed. Cells were treated with various compounds for 2h, rinsed, and then examined by confocal fluorescence microscopy. DIC and TAMRA images are shown. Nuclei with TAMRA fluorescence are noted with yellow arrows while ‘empty’ nuclei are noted with blue arrows. Controls (a, b); 10 µM UNC7938 (c,d); 10 µM UNC5103 (e,f); 30 µM UNC4954 (g,h).


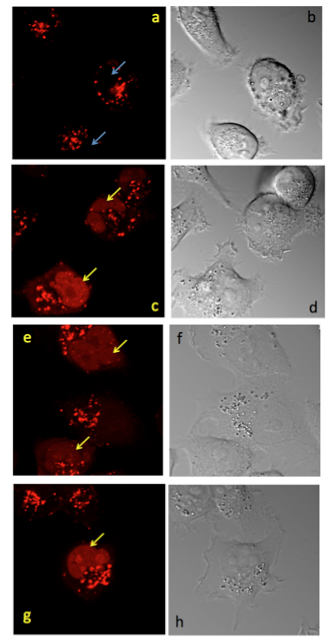


Supplementary Figure 4. *Effects of OECs on Organelle Morphology*. HeLa Luc 705 cells were incubated overnight with baculovirus vectors (50 u/cell) that express GFP chimeras of Rab7, LAMP1 and N-acetylgalactosaminyltransferase 2, which are marker proteins for late endosomes (LE), lysosomes (LY) or the Golgi (TG) respectively. The cells were rinsed and then treated for 2 h with 10 µM UNC7938 or UNC5103 or maintained as controls. Cells were then rinsed, fixed in 4% formaldehyde in PBS, and examined by confocal microscopy.


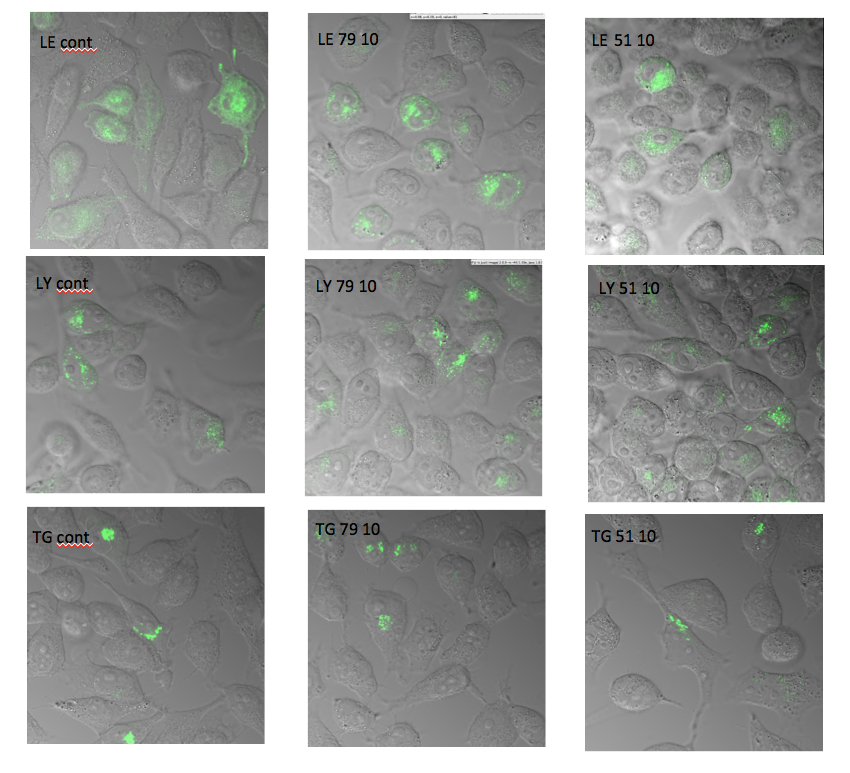


Supplementary Figure 5. *Lack of EGA Effects on Intracellular pH or Oligonucleotide Uptake*.

(a). *Intracellular pH*. Cells were incubated with 15 µM EGA or 300 µM chloroquine or maintained as controls for 15 min. At this point Lysotracker Red was added to a final concentration of 200 nM. After 20 min further incubation the cells were washed three times in PBS and Lysotracker accumulation measured using a plate reader with fluorescence detection.

(b) *Oligonucleotide Uptake*. Cells were pre-incubated for 15min with 15 µM EGA or maintained as controls. SSO623 labeled with a TAMRA fluorophore (623T) was added to a final concentration of 1 µM and the samples were incubated for 1h. Cells were washed 2 times in medium and 2 times in PBS, lysed, and TAMRA accumulation was measured using a plate reader with fluorescence detection. Cell protein was measured using the BCA method.


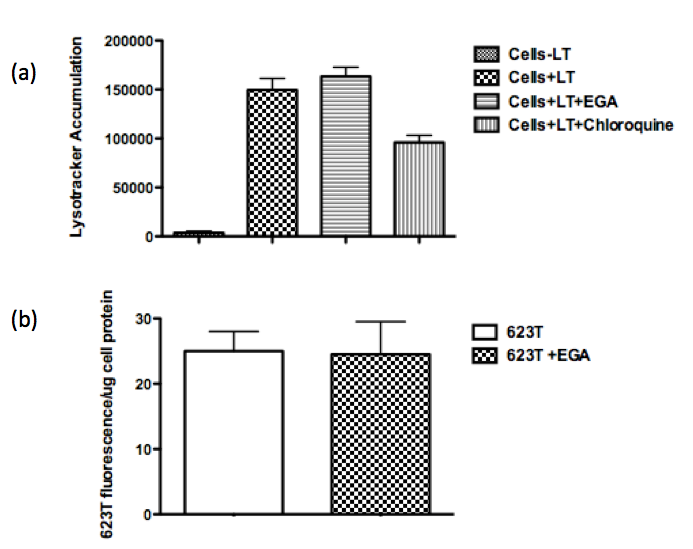


Supplementary Figure 6. *Effect of EGA on the Intracellular Distribution of a Fluorescent Oligonucleotide.* Cells were treated overnight with a baculovirus vector for expression of GFP-LAMP1, a lysosome marker protein. Cells were further incubated with SSO623-TAMRA in the presence or absence of 30 µM EGA for 1 h. The oligonucleotide was rinsed away and the incubation continued in the presence or absence of EGA using the environmental stage on the confocal microscope. Images were collected 15-30 min. after removal of the oligonucleotide.

The images depict the overlap of GFP-LAMP1 (green) and 623-TAMRA (red) with overlap areas in yellow/orange (indicated with arrows). Images (a) and (b) are of control cells while (c) and (d) are of EGA treated cells.


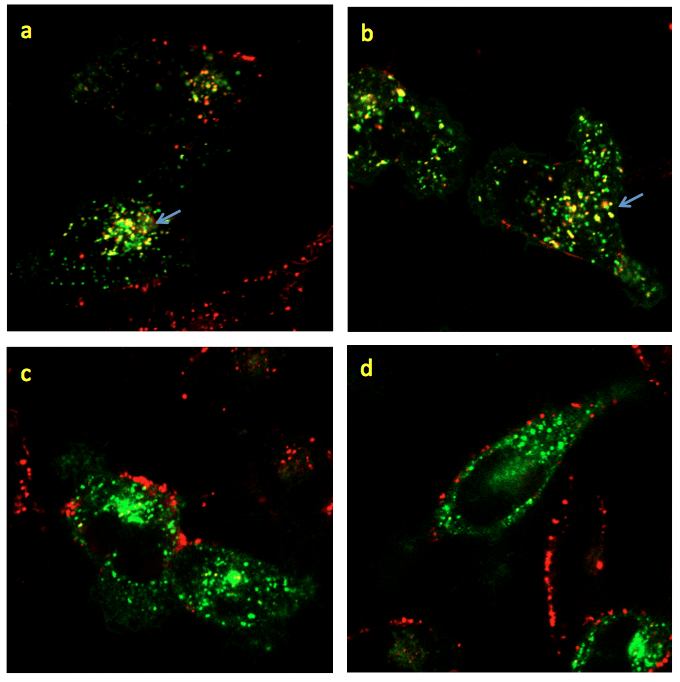


Supplementary Figure 7. *OEC Effects on Ethidium Dimer Uptake*. Cells were incubated in the presence of 4 µM ethidium dimer with co-incubation with various concentrations of the OECs UNC5103 or B-116. After 2h the cells were rinsed 1x in medium and 2x in PBS. Cells were lysed in 0.2% TX100 in and ethidium fluorescence measured on a plate reader. Cell protein was determined using the BCA method. A parallel experiment measured OEC cytotoxicity using the Alamar Blue assay.


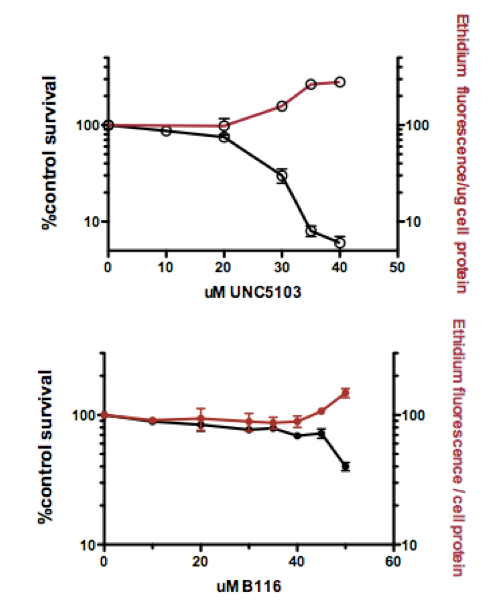

Supplement: Supplementary Data [file gkx1320_supp.zip › nar-03397-y-2017-File004.docx]
