# Supplementary material for: Structure–activity relationships and cellular mechanism of action of small molecules that enhance the delivery of oligonucleotides
Source: Nucleic Acids Res. 2018 Jan 18;46(4):1601–13. doi: 10.1093/nar/gkx1320 (PMC5829638; doi:10.1093/nar/gkx1320)

**SUPPLEMENTARY INFORMATION II.**

**Synthesis of UNC Compounds**

**General Information**

Analytical LCMS data for all compounds were acquired using an Agilent 6110 series system with the UV detector set to 220 and 254 nm. Samples were injected (<10 μL) onto an Agilent Eclipse Plus 4.6 × 50 mm, 1.8 um, C18 column at room temperature. A mobile phase of A (H_2_O + 0.1% acetic acid) and B (MeOH + 0.1% acetic acid) was used with a linear gradient from 10% to 100% B in 5.0 min, followed by a flush at 100% B for another 2 minutes with a flow rate of 1.0 mL/min. Mass spectra data were acquired in positive ion mode using an Agilent 6110 single quadrupole mass spectrometer with an electrospray ionization source. Nuclear Magnetic Resonance (NMR) spectra were recorded on a Varian Mercury spectrometer at 400 MHz for proton (^1^HNMR), and 100 MHz for carbon (^13^CNMR); chemical shifts are reported in ppm (δ). Analytical thin-layer chromatography (TLC) was performed with silica gel 60 F254, 0.25 mm pre-coated TLC plates, generally using a suitable MeOH in DCM solvent system. TLC plates were visualized using UV 254 nm, I_2_ impregnated silica gel, potassium permanganate with charring, and phosphomolybdic acid with charring. Reverse phase or normal phase chromatography was used to purify reaction mixtures to obtain intermediate products using a Teledyne Isco CombiFlash Rf 200 chromatography unit equipped with a UV detector set to 220 nm and 254 nm. Suitable variations in the purification method (flow rate, solvent system) were made as needed to achieve ideal separation for each compound. All compounds that were evaluated in biochemical and biophysical assays had >95% purity as determined by ^1^HNMR and LCMS.

Scheme 1. Example: Synthesis of **UNC5059**

**Intermediate 1** was synthesized according to the literature (Markees, D. G.; Kidder, G. W.

*J. Am. Chem. Soc. 78* (**1956**) 4130-5).

**Synthesis of Intermediate 2**

To a solution of intermediate 1 (680 mg, 2.36 mmol, 1.0 eq.) in H_2_SO_4_ (10 mL) was added KNO_3_ (285 mg, 2.8 mmol, 1.2 eq.) at 0°C and the reaction mixture was stirred at rt for 1h. Upon completion, the reaction was quenched by addition of 20 mL of water. The aqueous phase was extracted with EtOAc (3×20 mL), and the organic layers were combined, washed with brine, dried over anhydrous Na_2_SO_4_, filtered, and concentrated under reduced pressure to obtain a yellow crude material. The crude material was adsorbed onto silica gel, and purified by normal phase automated Teledyne Isco chromatography using a CH_2_Cl_2_/MeOH/NH_3_ solvent system. Intermediate 2 was obtained as pale yellow oil (395 mg, 50%).

^1^H NMR (400 MHz, DMSO-*d*_6_) δ 10.84 (brs, 1H), 10.52 (brs, 1H), 7.87 (s, 1H), 4.17 (q, *J* = 6.8 Hz, 2H), 4.07 (q, *J* = 7.1 Hz, 2H), 1.23 (t, *J* = 7.0 Hz, 3H), 1.17 (t, *J* = 7.1 Hz, 3H).

LC-MS (λ = 254 nm): 92%, t_R_ = 5.5 min. MS (ESI+): 333 [M+H]^+^

**Synthesis of Intermediate 3**

Water (0.7 mL) was added to a solution of Intermediate 2 (674 mg, 2.02 mmol, 1.0 eq.) in TEA (6.7 mL) at rt. The reaction mixture was stirred at 70°C overnight. The reaction was quenched by addition of 20 mL of water. The aqueous phase was extracted with EtOAc (3×20 mL), and the organic layers were combined, washed with brine, dried over anhydrous Na_2_SO_4_, filtered, and concentrated under reduced pressure to obtain a pure yellow product which was used in the next reaction without further purification (506 mg, 96%).

^1^H NMR (400 MHz, DMSO-*d*_6_) δ 10.48 (brs, 1H), 7.31 (s, 1H), 4.16 (q, *J* = 7.1 Hz, 2H), 1.24 (t, *J* = 7.1 Hz, 3H).

LC-MS (λ = 254 nm): 92%, t_R_ = 5.3 min. MS (ESI+): 261 [M+H]^+^

**Synthesis of Intermediate 4**

To a solution of intermediate 3 (100 mg, 0.38 mmol, 1.0 eq.) in EtOH (6.8 mL) and water (2.3 mL) was added Fe (107 mg, 1.92 mmol, 5.0 eq.) and NH_4_Cl (103 mg, 1.92 mmol, 5.0 eq.) at rt and the reaction mixture was heated at 90°C for 1h. Upon completion, the reaction was filtered through celite and washed with EtOH (50 mL). The filtrate was concentrated to obtain a greenish solid, which was then immediately used in the next reaction without further purification .

LC-MS (λ = 254 nm): 90%, t_R_ = 4.0 min. MS (ESI+): 231 [M+H]^+^

**Synthesis of Intermediate 5**

To a solution of crude intermediate 4 (340 mg, 1.47 mmol, 1.0 eq.) in EtOH (4 mL) and DMF (8 mL) was added benzil (372 mg, 1.77 mmol, 1.2 eq.) at rt and the reaction mixture was stirred at 100°C overnight. Upon completion, the reaction mixture was concentrated under reduced pressure to obtain a yellow crude material. The crude material was adsorbed onto silica gel, and purified by reverse phase automated Teledyne Isco chromatography using a CH_3_CN/H_2_O solvent system. Intermediate 5 was obtained as pale yellow solid (228 mg, 34%) over two steps .

^1^H NMR (400 MHz, DMSO-*d*_6_) δ 11.17 (brs, 1H), 8.60 (s, 1H), 7.50 (td, *J* = 7.5, 6.8, 1.6 Hz, 4H), 7.45 – 7.34 (m, 6H), 4.24 (q, *J* = 7.1 Hz, 2H), 1.29 (t, *J* = 7.1 Hz, 3H).

LC-MS (λ = 254 nm): 95%, t_R_ = 6.4 min. MS (ESI+): 405 [M+H]^+^

**Synthesis of UNC5059**

To a solution of intermediate 5 (50 mg, 0.12 mmol, 1.0 eq.) in 1,4 dioxane (2.0 mL) in a microwave vial was added 1-(2-aminoethyl)pyrrolidine (17 mg, 0.15 mmol, 1.2 eq.), and ^t^BuONa (23 mg, 0.24 mmol, 2.0 eq.). Argon gas was purged through the septum and the reaction was degassed for about 5 min. To the reaction mixture, DavePhos (4.7 mg, 0.012 mmol, 0.1 eq.) and Pd_2_(dba)_3_ (11 mg, 0.012 mmol, 0.1 eq.) were added and reaction mixture was purged with argon gas again for 5 min. The microwave vial was sealed and irradiated at 100°C for 1 h. The reaction mixture was filtered, washed with EtOAc (20 mL) and the filtrate was concentrated under reduced pressure. The crude material was adsorbed onto silica gel, and purified by reverse phase automated Teledyne Isco chromatography using a CH_3_CN/H_2_O solvent system. The product obtained was dissolved in DCM and washed with saturated aqueous solution of NaHCO_3_. The organic layer was washed with brine, dried over anhydrous Na_2_SO_4_, filtered, and concentrated under reduced pressure to obtain a yellow oil which was subsequently stirred with 2 mL of 4M HCl in 1,4-dioxane to obtain the HCL salt of UNC5059 as a pale yellow solid upon solvent evaporation (24 mg, 41%).

^1^H NMR (400 MHz, Chloroform-*d*) δ 12.40 (brs, 1H), 9.10 (d, *J* = 6.4 Hz, 1H), 7.67 (s, 1H), 7.57 (dd, *J* = 7.1, 1.6 Hz, 2H), 7.55 – 7.50 (m, 2H), 7.47 – 7.40 (m, 1H), 7.40 – 7.30 (m, 4H), 4.39 (q, *J* = 7.1 Hz, 2H), 4.12 (q, *J* = 6.2 Hz, 2H), 3.82 – 3.69 (m, 2H), 3.59 (t, *J* = 6.0 Hz, 2H), 3.11 - 2.99 (m, 2H), 2.14 – 2.02 (m, 4H), 1.40 (t, *J* = 7.1 Hz, 3H).

LC-MS (λ = 254 nm): 99%, t_R_ = 5.2 min. MS (ESI+): 484 [M+H]^+^

***UNC5103, UNC5127****, and* ***UNC5163*** *were prepared by a similar method as* ***UNC5059*** *for intermediate 5.*

**UNC5103**

Procedure for the synthesis of **UNC5059** was followed to obtain a yellow solid.

^1^H NMR (400 MHz, Chloroform-*d*) δ 8.23 (brs, 1H), 7.58 – 7.46 (m, 5H), 7.46 – 7.29 (m, 6H), 4.37 (q, *J* = 7.1 Hz, 2H), 3.78 – 3.58 (m, 2H), 3.34 – 3.16 (m, 2H), 2.81 (s, 6H), 2.33 – 2.17 (m, 2H), 1.39 (t, *J* = 7.1 Hz, 3H).

LC-MS (λ = 254 nm): 99%, t_R_ = 5.2 min. MS (ESI+): 472 [M+H]^+^

**UNC5127**

Procedure for the synthesis of **UNC5059** was followed to obtain a yellow solid.

^1^H NMR (400 MHz, DMSO-*d*_6_) δ 8.28 (brs, 1H), 7.58 – 7.50 (m, 2H), 7.51 – 7.42 (m, 2H), 7.47 – 7.32 (m, 6H), 7.31 (s, 1H), 4.24 (q, *J* = 7.1 Hz, 2H), 4.04 – 3.92 (m, 2H), 3.92 – 3.82 (m, 2H), 3.83 – 3.71 (m, 2H), 3.65 – 3.46 (m, 2H, overlapped), 3.23 – 3.15 (m, 2H), 1.29 (t, *J* = 7.1 Hz, 3H).

LC-MS (λ = 254 nm): 99%, t_R_ = 5.4 min. MS (ESI+): 499 [M+H]^+^

**UNC5163**

Procedure for the synthesis of **UNC5059** was followed to obtain a yellow solid.

^1^H NMR (400 MHz, Methanol-*d*_4_) δ 7.61 – 7.53 (m, 4H), 7.52 – 7.39 (m, 2H), 7.43 – 7.34 (m, 4H), 6.69 (s, 1H), 4.43 (q, *J* = 7.2 Hz, 2H), 3.94 (t, *J* = 6.2 Hz, 2H), 3.38 (t, *J* = 6.2 Hz, 2H), 1.42 (t, J = 7.1 Hz, 3H).

LC-MS (λ = 254 nm): 99%, t_R_ = 5.1 min. MS (ESI+): 429 [M+H]^+^


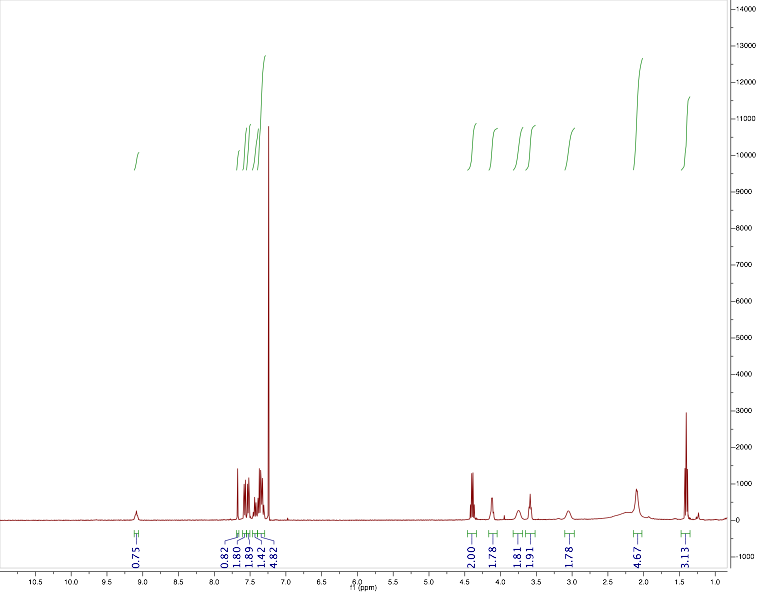


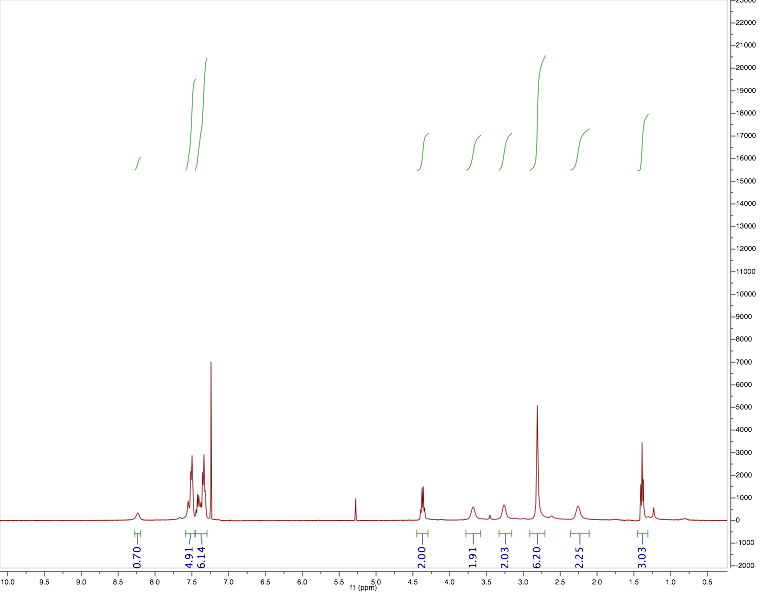


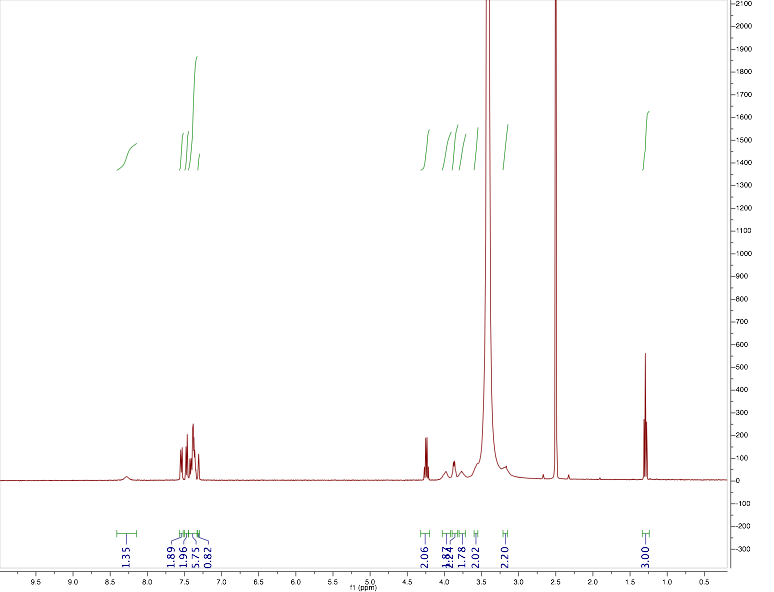


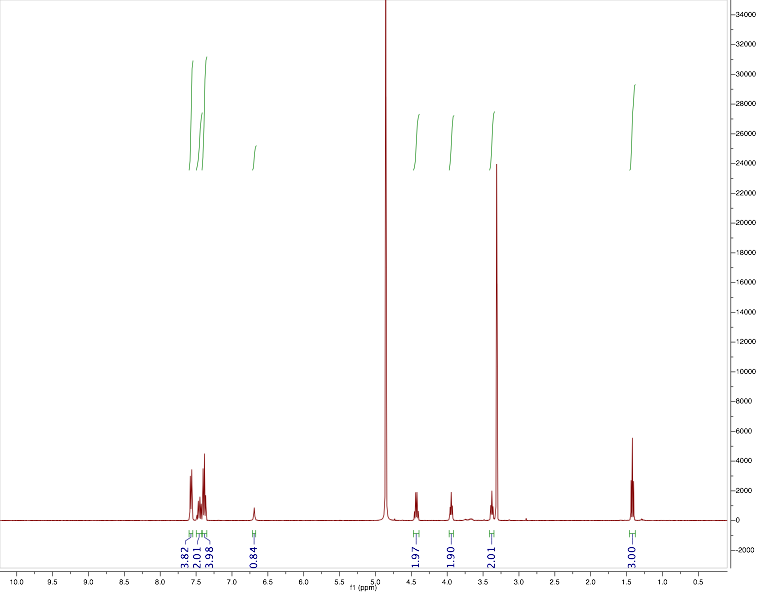


Scheme 2. Synthesis of **UNC4954**

Synthesis of **UNC4954**

To a solution of UNC10217938 (20 mg, 0.04 mmol) in EtOH (1 mL) and H_2_O (1 mL) was added KOH (100 mg) at rt and the reaction mixture was stirred at 100°C overnight. Upon completion, the precipitated product was filtered and washed with water. The solid was dissolved in MeOH and adsorbed onto silica gel, and purified by normal phase automated Teledyne Isco chromatography using a CH_2_Cl_2_/MeOH/NH_3_ solvent system. UNC4954 was obtained as a yellow solid (14 mg, 90%).

^1^H NMR (400 MHz, Methanol-*d*_4_) δ 7.51 – 7.40 (m, 4H), 7.39 – 7.24 (m, 6H), 6.01 (s, 1H), 3.47 (t, *J* = 6.6 Hz, 2H), 2.69 (t, *J* = 6.7 Hz, 2H), 2.34 (s, 6H).

LC-MS (λ = 254 nm): 99%, t_R_ = 4.3 min. MS (ESI+): 386 [M+H]^+^


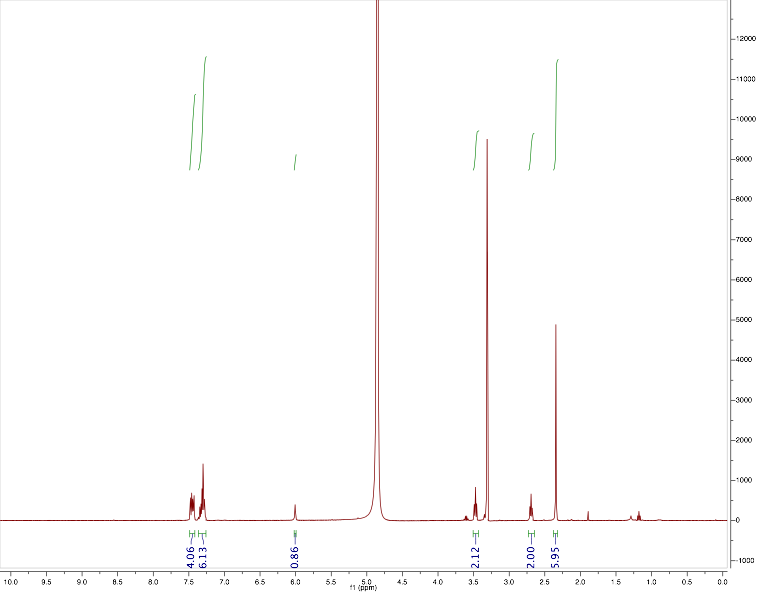

Supplement: Supplementary Data [file gkx1320_supp.zip › nar-03397-y-2017-File005.docx]
