# Supplementary material for: Structure–activity relationships and cellular mechanism of action of small molecules that enhance the delivery of oligonucleotides
Source: Nucleic Acids Res. 2018 Jan 18;46(4):1601–13. doi: 10.1093/nar/gkx1320 (PMC5829638; doi:10.1093/nar/gkx1320)
Supplement: Supplementary Data [file gkx1320_supp.zip › SUPPLEMENTARY_INFORMATION.docx]

**SUPPLEMENTARY INFORMATION III**

**Synthesis of Chemogenics Biopharma Compounds**


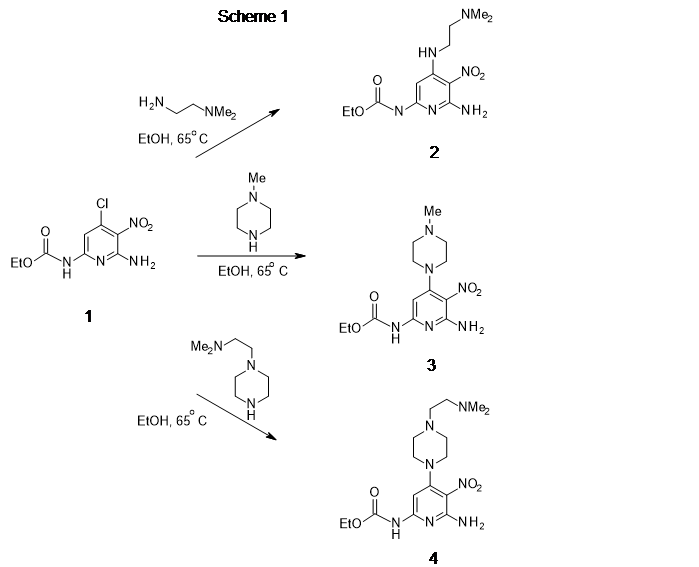


In Scheme 1, intermediate 1 was synthesized according to literature procedures. Intermediate 1 was treated with various amines in ethanol as solvent to afford the desired compounds 2-4.


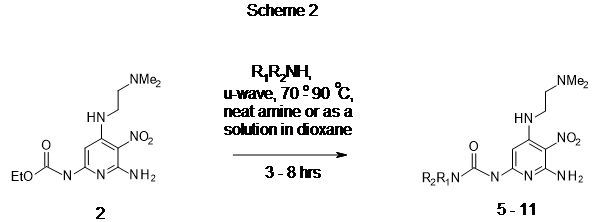


Compound 2 was converted to the urea derivatives as in scheme 2 to afford compounds 5-11.


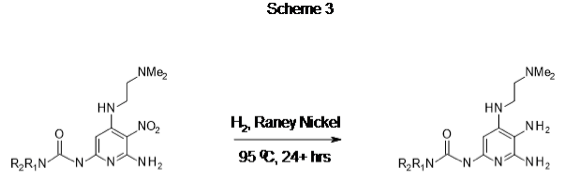


**5-11 16-22**

Compounds 5 – 11 were treated with Raney Nickel in presence of hydrogen to afford the diamines 16 -22 as in scheme3.


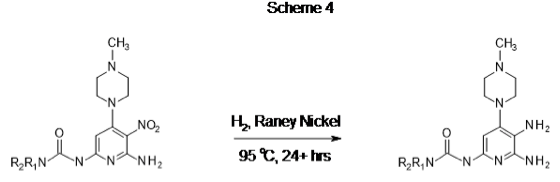


**12-13 23-24**

Compounds 12 – 13 were also converted to the diamines using the Raney Nickel reduction conditions to afford compounds 23-24.


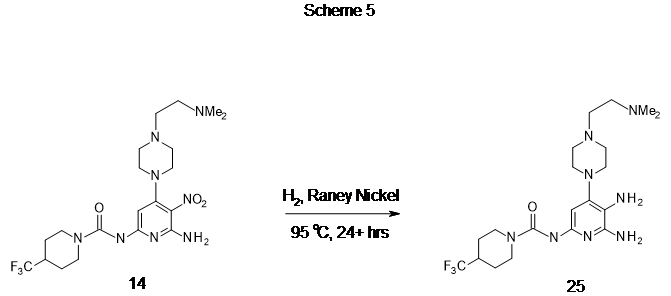


The final targets were made using the general method as in Scheme 6 below


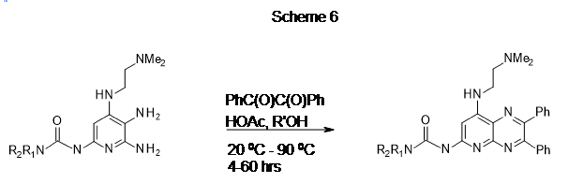


**16-22**

**General Experimental:**

Starting with chelidamic acid **A**, (4-chloropyridine-2,6-diyl)carbonyl dichloride **B**,

dimethyl 4-chloropyridine-2,6-dicarboxylate **C**, (4-chloropyridine-2,6-diyl)carboxylic acid dihydrazide **D**, (4-chloropyridine-2,6-diyl)carboxylic acid diazide **E**, diethyl (4-chloropyridine-2,6-diyl)bis[carbamate] **F**, diethyl (4-chloro-3-nitropyridine-2,6-diyl)bis[carbamate] **G**, and ethyl (6-amino-4-chloro-5-nitropyridine-2-yl) carbamate **1**, were synthesized via procedures described or cited by Temple, Jr., C.; Rener, G. A. and Comber, R. N. in *J. Med.Chem.,* **1989**, *32* (10), pp 2363–2367. Purifications using MPLC were performed using an ISCO Foxy Jr., instrument equipped with an ISCO UA6 UV/vis detector and SiliaSep^TM^ silica gel columns purchased from Silicycle. Microwave-assisted reactions were performed using a CEM Discover/Explorer instrument operating with Synergy software controls. NMR spectra were determined on DMSO-d_6_ or CDCl_3_ solutions using a Bruker 500 MHz spectrometer with solvent as the internal reference at 2.50 ppm (DMSO-d_5_) or 7.26 ppm (CHCl_3_). Mass spectra were taken with a PE SCIEX API 100 spectrometer using fast atom bombardment mode to provide the (M + 1)^+^ molecular ion. The progress of reactions was followed by thin-layer chromatography (TLC) on plates of silica gel from Analtech, Inc., or using HPLC analysis using a Supelco Ascentis^®^ 15cm x 4.6 mm x 5 um Phenyl column on a Shimadzu SIL-HTC instrument (eluent starting ratio of 98%: 2:: 0.05% TFA in water: methanol using a gradient to 100% MeOH over 16 minutes; held at that percentage of methanol until 20 minutes with a flow rate of 1.2 mL/min). HPLC detection of peaks was by UV absorbance between 190 and 450 nm) using a Dynamax PDA-2 diode array detector. Solvents and reagents were obtained from commercial sources and were used as received.

**Ethyl N-(6-amino-4-[2-dimethylaminoethylamino]-5-nitropyridine-2-yl) carbamate 2**. The following procedure can be considered to be a general procedure for the synthesis of ethyl 6-amino-4-alkylamino-5-nitropyridine-2-yl) carbamate derivatives such as **2**:

To a suspension of **1** (520 mg, 2.00 mmol) in absolute ethanol (4.0 mL) at room temperature was added dimethylaminoethyl amine (441 mg, 5.0 mmol). The flask was flushed with N_2_ and sealed with a septum attached via a needle to an N_2_ source. The reaction was then placed in an oil bath at 65-70° C and was monitored by TLC (99: 1 :: CH_2_Cl_2_: MeOH). The reaction changed from yellow to deep orange over time as **1** dissolved and formed **2**. After 4 hr reaction, TLC indicated an absence of **1** so the solvent was removed *i.v*. using a rotary evaporator. The residue was purified by MPLC by dissolving it in 5 mL of CH_2_Cl_2_ and loading onto a 40g column (flow rate = 40 mL/min; starting eluent = 100% CH_2_Cl_2_ with a gradient to 5% MeOH: CH_2_Cl_2_ over 30 min, then to a second gradient of 3% conc. NH_4_OH: 20% MeOH; 77% CH_2_Cl_2_ over 10 min. Gave **9** (564 mg, (90.2% yield) as a bright yellow powder. ^1^H NMR (500 MHz, CHLOROFORM-*d*) δ ppm 1.31 (t, *J*=7.09 Hz, 3 H) 2.31 (s, 6 H) 2.64 (t, *J*=6.11 Hz, 2 H) 3.36 (q, 2 H) 4.23 (q, *J*=7.34 Hz, 2 H) 6.77 (s, 1 H) 9.33 (br. s., 1 H) 12.63 (s, 1H). MS: (M + 1)^+^: 313.

**Ethyl N-(6-amino-4-[4-methylpiperazine-1-yl]-5-nitropyridine-2-yl) carbamate 3**. Using a procedure similar to that for the synthesis of **2**, compound **1**

(400 mg, 1.54 mmol) was converted into **3** using N-methylpiperazine (384 mg, 3.84 mmol) in absolute ethanol (5.0 mL). After 3 hr, TLC indicated an absence of **1**, so the solvent was removed *i.v.* by rotary evaporation and the residue was purified by MPLC to give **3** (467 mg, 93.8% yield) as a yellow powder. ^1^H NMR (500 MHz, CHLOROFORM-*d*) δ ppm 1.31 (t, *J*=7.34 Hz, 3 H) 2.33 (s, 3 H) 2.44 - 2.68 (m, 4 H) 3.15 - 3.37 (m, 4 H) 4.23 (q, *J*=6.85 Hz, 2 H) 6.48 (br. s., 2 H) 6.97 (s, 1 H) 7.97 (br. s., 1 H). MS: (M + 1)^+^: 325.

**Ethyl N-(6-amino-4-[4-{2-dimethylaminoethyl}piperazin-1-yl]-5-nitropyridine-2-yl) carbamate 4**. Using a procedure similar to that for the synthesis of **2**, compound **1** (300 mg, 1.15 mmol) was converted into **4** using 1-(2-dimethylaminoethyl)-piperazine (453 mg, 2.88 mmol) in absolute ethanol (3.0 mL). Gave a yellow precipitate from the crude ethanol solution when cooled to room temperature, so the suspension was further cooled in the freezer, then suction filtered and rinsed with a little ethanol to give **4** (375 mg, 85.4% yield) as a bright yellow powder after air drying. The filtrate could be purified by MPLC, if desired. ^1^H NMR (500 MHz, CHLOROFORM-*d*) δ ppm 1.32 (t, *J*=7.09 Hz, 3 H) 2.30 (s, 6 H) 2.51 (t, 2 H) 2.54 - 2.59 (m, 2 H) 2.59 - 2.65 (m, 4 H) 3.21 - 3.32 (m, 4 H) 4.23 (q, *J*=7.34 Hz, 2 H) 6.41 (br. s., 2 H) 6.97 (s, 1 H) 7.58 (br. s., 1 H). MS: (M + 1)^+^: 382.

**General procedure for the synthesis of urea derivatives like 5:**

A solution of the appropriate carbamate (e.g., ethyl N-(6-amino-4-[2-dimethylaminoethylamino]-5-nitropyridine-2-yl) carbamate **2**) was treated with a large excess of the required amine either as a neat mixture or as a solution in anhydrous 1,4-dioxane in a microwave reaction vessel. The vessel was flushed with nitrogen gas and the sealed mixture was stirred with a magnetic stirbar while heating in the microwave machine at 140° C under pressure for several hours. When no carbamate was present by TLC analysis, the reaction mixture was transferred into a round bottom flask and the volatiles were removed using a rotary evaporator. The residue of crude urea was purified either by trituration with ether to remove any residual amine starting material or by MPLC using a gradient from 100% CH_2_Cl_2_ to 100% x (1:14:85 :: conc NH_4_OH: MeOH: CH_2_Cl_2_) on a 12g silica gel column).

**Morpholin-4-yl N-[6-amino-4-[2-(dimethylamino)ethylamino]-5-nitro-2-pyridyl] urea 5.**

A solution of carbamate **2** (100 mg, 0.320 mmol) in morpholine (900 mg, 10.3 mmol) was prepared in a microwave reaction vessel containing a magnetic stirbar. The vessel was flushed with nitrogen, sealed with a pressure-tight cap and placed in the microwave apparatus. Stirring was started and the reaction was heated to 140° C under pressure for 5 hours at which point no **2** remained by TLC analysis. The morpholine was removed *i.v.* and the residue began to solidify after most of the morpholine had been removed. Trituration with ether (2 x 3 mL), filtration and air-drying gave **5** (107 mg, 94.5% yield) as a yellow powder. ^1^H NMR (599 MHz, CHLOROFORM-*d*) δ ppm 2.30 (s, 6 H) 2.56 - 2.65 (m, 2 H) 3.30 - 3.37 (m, 2 H) 3.47 - 3.53 (m, 4 H) 3.71 - 3.78 (m, 4 H) 6.88 (s, 1 H) 6.98 (br. s., 1 H) 9.36 (br. s., 2 H). MS: (M + 1)^+^: 354.

**Piperidin-1-yl N-[6-amino-4-[2-(dimethylamino)ethylamino]-5-nitro-2-pyridyl] urea 6**.

A solution of carbamate **2** (200 mg, 0.640 mmol) in piperidine (1.55 g, 18.2 mmol) gave **6** (157 mg, 69.8% yield) as a yellow powder after microwave reaction, concentration *i.v.*, ether triturations and air-drying as provided in the general experimental. ^1^H NMR (500 MHz, CHLOROFORM-d) δ ppm 1.56 - 1.71 (m, 6 H) 2.28 (s, 6 H) 2.60 (t, J=6.11 Hz, 2 H) 3.30 - 3.39 (m, 2 H) 3.43 - 3.50 (m, 4 H) 6.79 – 7.25 (b. s., 2 H) 6.91 (s, 1 H) 6.95 (br. s., 1 H) 9.29 (br. s., 1 H). MS: (M + 1)^+^: 352.

**4-Methylpiperazine-1-yl N-[6-amino-4-[2-(dimethylamino)ethylamino]-5-nitro-2-pyridyl] urea 7**.

A solution of carbamate **2** (78 mg, 0.25 mmol) in 4-methylpiperazine (1.11 g, 11.1 mmol) gave **7** (62 mg, 67.7% yield) as a yellow powder after microwave reaction, concentration *i.v.*, and purification by MPLC as provided in the general experimental. ^1^H NMR (500 MHz, CHLOROFORM-d) δ ppm 2.28 (s, 6 H) 2.33 (s, 3 H) 2.41 - 2.47 (m, 4 H) 2.60 (t, J=6.11 Hz, 2 H) 3.34 (dd, 2 H) 3.49 - 3.57 (m, 4 H) 5.51 – 8.46 (b. s., 2 H) 6.89 (s, 1 H) 6.98 (br. s., 1 H) 9.30 (br. s., 1 H). MS: (M + 1)^+^: 367.

**N-Phenyl N’-[6-amino-4-[2-(dimethylamino)ethylamino]-5-nitro-2-pyridyl] urea 8**.

A solution of carbamate **2** (103 mg, 0.330 mmol) in aniline (1.02 g, 11.0 mmol) gave **8** (84 mg, 70.8% yield) as a yellow powder after microwave reaction, concentration *i.v.*, ether triturations and air-drying as provided in the general experimental. MS: (M + 1)^+^: 360.

**4-(Trifluoromethyl)piperidine-1-yl) N-[6-amino-4-[2-(dimethylamino)ethylamino]-5-nitro-2-pyridyl] urea 9**.

A solution of carbamate **2** (200 mg, 0.640 mmol) in 4-trifluoromethylpiperidine (278 mg, 1.81 mmol) and anhydrous 1,4-dioxane (2.0 mL) gave **9** (132 mg, 49.1% yield) as a yellow powder after microwave reaction, concentration *i.v.*, ether triturations and air-drying as provided in the general experimental. ^1^H NMR (500 MHz, CHLOROFORM-d) δ ppm 1.50 - 1.69 (m, J=12.72, 12.72, 12.47, 4.16 Hz, 2 H) 1.87 - 2.05 (m, 3 H) 2.26 (s, 6 H) 2.60 (t, J=6.11 Hz, 2 H) 2.90 (t, J=12.96 Hz, 2 H) 3.27 - 3.39 (m, 2 H) 4.19 (d, J=13.69 Hz, 2 H) 6.86 (s, 1 H) 7.05 (br. s., 1 H) 9.31 (br. s., 1 H). MS: (M + 1)^+^: 420.

**4,4-Dimethylpiperidine-1-yl N-[6-amino-4-[2-(dimethylamino)ethylamino]-5-nitro-2-pyridyl] urea 10.**

A solution of carbamate **2** (243 mg, 0.778 mmol) and 4,4-dimethylpiperidine (377 mg, 3.34 mmol) in anhydrous 1,4-dioxane (1.0 mL) gave **10** (101 mg, 34.2% yield) as a yellow foam after microwave reaction, concentration *i.v.*, and MPLC as provided in the general experimental. ^1^H NMR (500 MHz, CHLOROFORM-d) δ ppm 1.00 (s, 6 H) 1.36 - 1.48 (m, 4 H) 2.29 (s, 6 H) 2.60 (t, J=6.11 Hz, 2 H) 3.29 - 3.39 (m, 2 H) 3.41 - 3.50 (m, 4 H) 6.92 (s, 1 H) 6.98 (br. s., 1 H) 9.31 (br. s., 1 H). MS: (M + 1)^+^: 380.

**3,5-Dimethylpiperidine-1-yl N’-[6-amino-4-[2-(dimethylamino)ethylamino]-5-nitro-2-pyridyl] urea 11**.

A solution of carbamate **2** (80 mg, 0.256 mmol) and 3,5-dimethylpiperidine (64 mg, 0.566 mmol) in anhydrous 1,4-dioxane (1.0 mL) gave **11** (101 mg, 34.2% yield) as a yellow powder after microwave reaction (8 hr) and concentration *i.v.*, as provided in the general experimental and using crystallization from MeOH at -20 C to purify the product instead of ether triturations. ^1^H NMR (500 MHz, CHLOROFORM-d) δ ppm 0.72 - 0.82 (q, 1 H) 0.93 (d, J=6.36 Hz, 6 H) 1.54 - 1.71 (m, 2 H) 1.80 - 1.91 (m, 1 H) 2.28 (s, 6 H) 2.32 - 2.40 (m, 2 H) 2.60 (t, J=5.87 Hz, 2 H) 3.30 - 3.39 (m, 2 H) 3.98 (d, J=11.25 Hz, 2 H) 6.90 (br. s., 1 H) 6.92 (s, 1 H) 9.32 (br. s., 1 H). MS: (M + 1)^+^: 380.

**Piperidine-1-yl N’-[6-amino-4-(4-methylpiperazin-1-yl)-5-nitro-2-pyridyl] urea 12**.

A solution of carbamate **3** (100 mg, 0.446 mmol) in piperidine (393 mg, 4.56 mmol) gave **12** (75 mg, 46.3% yield) as a yellow foam after microwave reaction, concentration *i.v.*, and MPLC purification as provided in the general experimental. ^1^H NMR (500 MHz, CHLOROFORM-d) δ ppm 1.53 - 1.72 (m, 6 H) 2.33 (s, 3 H) 2.45 - 2.59 (m, 4 H) 3.21 - 3.31 (m, 4 H) 3.39 - 3.52 (m, 4 H) 6.30 (br. s., 2 H) 6.99 (s, 1 H) 7.12 (s, 1 H). MS: (M + 1)^+^: 364.

**4-(Trifluoromethyl)piperidine-1-yl N’-[6-amino-4-(4-methylpiperazin-1-yl)-5-nitro-2-pyridyl] urea 13**.

A solution of carbamate **3** (150 mg, 0.669 mmol) in 4-trifluoromethylpiperidine (512 mg, 3.34 mmol) gave **13** (117 mg, 40.6% yield) as a yellow foam after microwave reaction, concentration *i.v.*, and MPLC purification as provided in the general experimental. ^1^H NMR (500 MHz, CHLOROFORM-d) δ ppm 1.53 - 1.66 (m, 2 H) 1.98 (d, J=11.25 Hz, 2 H) 2.19 - 2.32 (m, 1 H) 2.34 (s, 3 H) 2.48 - 2.60 (m, 4 H) 2.93 (t, 2 H) 3.22 - 3.30 (m, 4 H) 4.18 (d, J=13.21 Hz, 2 H) 6.26 (br. s., 2 H) 6.92 (br. s., 1 H) 7.09 (s, 1 H). MS: (M + 1)^+^: 432.

**4-(Trifluoromethyl)piperidine-1-yl N’-[6-amino-4-[4-[2-(dimethylamino)ethyl]piperazin-1-yl]-5-nitro-2-pyridyl] urea 14**.

A solution of carbamate **4** (200 mg, 0.525 mmol) and 4-trifluoromethylpiperidine (243 mg, 1.59 mmol) in anhydrous 1,4-dioxane (2.0 mL) gave **14** (123 mg, 48.0% yield) as a yellow foam after microwave reaction, concentration *i.v.*, and MPLC purification as provided in the general experimental. ^1^H NMR (500 MHz, CHLOROFORM-d) δ ppm 1.59 (qd, J=12.72, 4.40 Hz, 2 H) 1.85 (br. s., 3 H) 1.96 (d, J=13.69 Hz, 2 H) 2.25 (s, 6 H) 2.45 (t, J=6.60 Hz, 2 H) 2.48 - 2.55 (m, 2 H) 2.55 - 2.63 (m, 4 H) 2.83 - 2.96 (m, 2 H) 3.18 - 3.30 (m, 4 H) 4.18 (d, J=12.23 Hz, 2 H) 6.27 (br. s., 2 H) 7.00 (br. s., 1 H) 7.07 (s, 1 H). MS: (M + 1)^+^: 489.

**Synthesis of dialkyl diketones:**

**Decane-5,6-dione.**

To a stirring solution of valeroin (*i.e*., 6-hydroxy-5-octanone; 1.00 mL; 5.28 mmol) in CH_2_Cl_2_ (11 mL) was added Dess-Martin Periodinane reagent (2.46 g; 5.81 mmol). The flask was flushed with nitrogen gas and sealed with a septum under N_2_ via a needle and nitrogen line. The reaction became warm to the touch. The mixture was stirred at room temperature for an hour and then a solution of 20 mL water, 20 mL aq. saturated NaHCO_3_ solution and 40 mL of 10% aq. sodium thiosulfate was added; the mixture was stirred for 5 min, then CH_2_Cl_2_ (20 mL) was added and the layers were separated. The aq. portion was extracted with CH_2_Cl_2_ (20 mL) and the organic layers were combined and dried with MgSO_4_. The solvent was removed *i.v.* on the rotovap to give a yellow oil. This material was purified by filtration through a plastic syringe barrel containing a small plug of cotton at the needle end of the barrel and 15 mL of silica gel. The eluent used in the filtration was 25% CH_2_Cl_2_/ hexane. The eluted solution was concentrated by rotary evaporation to give **Decane-5,6-dione** (759 mg; 83.4% yield). ^1^H NMR (500 MHz, CHLOROFORM-d) δ ppm 0.92 (t, J=7.34 Hz, 6 H) 1.28 - 1.39 (m, J=15.04, 7.46, 7.34, 7.34 Hz, 4 H) 1.51 - 1.62 (m, 4 H) 2.74 (t, J=7.34 Hz, 4 H).

**1,2-Dicyclohexylethanedione.**

Used the procedure of Babudri, et al. [*Tetrahedron. Lett.* **36** (40), 7305-7308, 1995] with modifications for the synthesis of diketones below. The following will act a general example of the procedure used:

Cuprous bromide (1.78 g; 12.4 mmol) was placed in an oven-dried round bottom flask containing a stirbar; the flask was flushed with dry N_2_ gas and was sealed using a septum connected via a needle to a nitrogen line. Anhydrous THF (45 mL) was added via syringe to give a suspension of solid. This was stirred at room temperature while a 1.5 M solution of LiBr in anhydrous THF (21.6 mL, 32.4 mmol) was added via syringe under nitrogen. The reaction stirred for 15 min during which time the CuBr dissolved to give a pale green solution. The mixture was cooled to -78° C in a dry ice/acetone bath for 10 min, then a ca. 1.0 M solution of cyclohexyl magnesium bromide in THF (12.4 mL, 12.4 mmol) was added to the solution over 2 min to give a yellow-brown solution. After 15 min, freshly-distilled oxalyl chloride (660 mg, 5.20 mmol) was added and the reaction was stirred for 5 min at -78° C. The reaction was quenched using a saturated aq. solution of ammonium chloride (10 mL) and letting the reaction come to room temperature. The solvent was removed *i.v.* using rotary evaporation and the residue was treated with CH_2_Cl_2_ (2 x 50 mL). The combined organic solution was dried over K_2_CO_3_, then was filtered and concentrated *i.v.* to give a yellow oil. The material was purified by MPLC using a silica gel column (40 g) and an eluent gradient system from 15% CH_2_Cl_2_/ hexane to 100% CH_2_Cl_2_. Gave **1,2-Dicyclohexylethanedione** (540 mg, 19.6% yield) as a yellow oil that solidified upon standing at room temperature. ^1^H NMR (599 MHz, CHLOROFORM-d) δ ppm 1.12 - 1.39 (m, 12 H) 1.74 - 1.83 (m, 8 H) 3.10 (tt, J=11.31, 3.18 Hz, 2 H).

**1,2-Dicyclopentylethanedione.**

Compound **1,2-Dicyclopentylethanedione** was synthesized using the above procedure from cuprous bromide (1.98 g, 13.8 mmol) in anhydrous THF (45 mL), 1.5 M LiBr in THF (18.0 mL, 27.6 mmol), 1.1 M cyclopentylmagnesium bromide in THF (11.3 mL, 12.4 mmol) and oxalyl chloride (823 mg, 6.49 mmol). The crude material was purified by MPLC using an eluent system from 5% CH_2_Cl_2_/ hexane to 100% CH_2_Cl_2_. Gave **1,2-Dicyclopentylethanedione** (181 mg, 14.4% yield) as a yellow oil. ^1^H NMR (599 MHz, CHLOROFORM-d) δ ppm 1.56 - 1.68 (m, 4 H) 1.79 - 1.92 (m, 4 H) 3.47 - 3.63 (m, 2 H).

**Tetradecane-7,8-dione.**

Compound **Tetradecane-7,8-dione** was synthesized using the above procedure from cuprous bromide (1.98 g, 13.8 mmol) in anhydrous THF (45 mL), 1.5 M LiBr in THF (18.0 mL, 27.6 mmol), 1.1 M hexylmagnesium bromide in THF (11.3 mL, 12.4 mmol) and oxalyl chloride (823 mg, 6.49 mmol). The crude material was purified by MPLC using a 40 g silica gel column and an eluent system from 10% CH_2_Cl_2_/ hexane to 100% CH_2_Cl_2_. Gave **Tetradecane-7,8-dione** (350 mg, 23.8% yield) as a yellow oil that solidified upon standing at room temperature. ^1^H NMR (500 MHz, CHLOROFORM-d) δ ppm 0.84 - 0.93 (m, 6 H) 1.10 - 1.36 (m, 12 H) 1.52 - 1.62 (m, 4 H) 2.73 (t, J=7.34 Hz, 4 H).

**Octadecane-9,10-dione.**

Compound **Octadecane-9,10-dione** was synthesized using the above procedure from cuprous bromide (1.98 g, 13.8 mmol) in anhydrous THF (45 mL), 1.5 M LiBr in THF (18.0 mL, 27.6 mmol), 1.1 M octylmagnesium bromide in THF (11.3 mL, 12.4 mmol) and oxalyl chloride (823 mg, 6.49 mmol). The crude material was purified by MPLC using a 40 g silica gel column and an eluent system from 5% CH_2_Cl_2_/ hexane to 100% CH_2_Cl_2_. Gave **Octadecane-9,10-dione** (915 mg, 49.9% yield) as a yellow oil that solidified upon standing at room temperature. ^1^H NMR (500 MHz, CHLOROFORM-d) δ ppm 0.88 (t, J=7.09 Hz, 6 H) 1.20 - 1.38 (m, 20 H) 1.53 - 1.61 (m, 4 H) 2.73 (t, J=7.34 Hz, 4 H).

**Ethyl N-(5,6-diamino-4-[2-dimethylaminoethylamino]pyridine-2-yl) carbamate 15**.

The following procedure will act as a general example for the reduction of the 5-nitropyridine derivatives to 5-aminopyridine derivatives:

A commercial sample of non-activated Raney Nickel (1g) was placed in an Erlenmeyer flask and was activated by treatment with 3M aq. HCl (20 mL). The suspension was heated until a gentle evolution of gas was noted, then was allowed to sit for 3 min to activate. The mixture was suction-filtered through a fritted glass filter and the solid residue was quickly rinsed on the filter in sequence with water (20 mL) and absolute ethanol (2 x 5 mL). The damp gray solid was placed in a round bottom flask containing a stirbar, a bright yellow solution of **2** (148 mg, 0.474 mmol) in absolute ethanol (8 mL) and glacial acetic acid (2 mL). The flask was flushed with nitrogen gas and placed, unsealed, into a stainless steel pressure bomb that was standing in an oil bath. The bomb was sealed and stirring was initiated using a magnetic stirrer. The bomb was evacuated under reduced pressure briefly and was filled with hydrogen gas to 400 psi pressure, then the oil bath was heated to 90° C. After 72 hours, the bomb was vented and then evacuated under reduced pressure. After allowing air into the bomb, it was unsealed and the reaction progress was evaluated by TLC and HPLC. The solution at this point was colorless and the analyses showed that no **2** remained. The mixture was suction filtered through a 2 cm pad of Celite on a fritted filter and the solid metal residue was washed with methanol (2 x 20 mL). The solvent was removed by rotary evaporation at reduced pressure to give an air-sensitive, brown oil which was used in crude form due to its tendency to decompose via air oxidation. The residue was dissolved in absolute ethanol (3.0 mL) and the solution of **15** was kept sealed and used under nitrogen. It was assumed that the yield of **15** was 100% and that the concentration of **15** in the solution was, therefore, 0.158 M. In the case of **15**, a small amount of this solution was concentrated on the rotovap and an NMR was taken. ^1^H NMR (599 MHz, DMSO- d_6_) δ ppm 1.11 (t, J=7.17 Hz, 3 H) 2.09 (s, 6 H) 2.38 (t, J=6.44 Hz, 2 H) 2.96 - 3.05 (m, 2 H) 3.92 - 4.01 (q, 2 H) 4.81 (br. s., 2 H) 6.44 (s, 1 H) 8.83 (br. s., 1 H). MS: (M + 1)^+^: 283.

**Morpholine-4-yl N’-[5,6-diamino-4-[2-(dimethylamino)ethylamino]-2-pyridyl] urea 16.**

The general procedure used to synthesize **15** from **2** was used similarly for the synthesis of highly air-sensitive urea **16** from **5**. ^1^H NMR (599 MHz, DMSO-d6) δ ppm 2.21 (s, 6 H) 2.80 - 2.91 (m, 2 H) 3.04 - 3.15 (m, 2 H) 3.34 - 3.45 (m, 4 H) 3.53 - 3.59 (m, 4 H) 3.66 (br. s., 2 H) 9.01-9.34 (br. s., 2 H). MS: (M + 1)^+^: 324.

**Piperidine-1-yl N’-[5,6-diamino-4-[2-(dimethylamino)ethylamino]-2-pyridyl] urea 17.**

The general procedure used to synthesize **15** could be used to produce the highly air-sensitive urea **17** from nitro compound **6**. This was used in crude form for the next reaction. MS: (M + 1)^+^: 322.

**4-Methylpiperazin-1-yl N’-[5,6-diamino-4-[2-(dimethylamino)ethylamino]-2-pyridyl] urea 18.**

The general procedure used to synthesize **15** could be used to produce the highly air-sensitive urea **18** from nitro compound **7**. This was used in crude form for the next reaction. MS: (M + 1)^+^: 385.

**N-phenyl N’-[5,6-diamino-4-[2-(dimethylamino)ethylamino]-2-pyridyl] urea 19.**

The general procedure used to synthesize **15** could be used to produce the highly air-sensitive urea **19** from nitro compound **8**. This was used in crude form for the next reaction. MS: (M + 1)^+^: 330.

**4-(trifluoromethyl)piperidine-1-yl N’-[5,6-diamino-4-[2-(dimethylamino)ethylamino]-2-pyridyl] urea 20.**

The general procedure used to synthesize **15** could be used to produce the highly air-sensitive urea **20** from nitro compound **9**. This was used in crude form for the next reaction. MS: (M + 1)^+^: 390.

**4,4-dimethylpiperidin-1-yl N’-(5,6-diamino-4-[2-dimethylaminoethylamino] pyridin-2-yl) urea 21.**

The general procedure used to synthesize **15** could be used to produce the highly air-sensitive urea **21** from nitro compound **10**. This was used in crude form for the next reaction. MS: (M + 1)^+^: 350.

**3,5-dimethylpiperidine-1-yl N’-[5,6-diamino-4-[2-(dimethylamino)ethylamino]-2-pyridyl] urea 22.**

The general procedure used to synthesize **15** could be used to produce the highly air-sensitive urea **22** from nitro compound **11**. This was used in crude form for the next reaction. MS: (M + 1)^+^: 350.

**Piperidine-1-yl N’-[5,6-diamino-4-(4-methylpiperazin-1-yl)-2-pyridyl] urea 23**

The general procedure used to synthesize **15** could be used to produce the highly air-sensitive urea **23** from nitro compound **12**. This was used in crude form for the next reaction. MS: (M + 1)^+^: 334.

**4-(Trifluoromethyl)piperidine-1-yl N’-[5,6-diamino-4-(4-methylpiperazin-1-yl)-2-pyridyl] urea 24.**

The general procedure used to synthesize **15** could be used to produce the highly air-sensitive urea **24** from nitro compound **13**. This was used in crude form for the next reaction. MS: (M + 1)^+^: 402.

**4-(Trifluoromethyl)piperidine-1-yl N’-[5,6-diamino-4-[4-[2-(dimethylamino)ethyl]piperazin-1-yl]-2-pyridyl] urea 25.**

The general procedure used to synthesize **15** could be used to produce the highly air-sensitive urea **25** from nitro compound **14**. This was used in crude form for the next reaction. MS: (M + 1)^+^: 459.

**Ethyl N-(2,3-dibutyl-8-[2-dimethylaminoethylamino]-1,4,5-triazanaphthalene-6-yl) carbamate (B1-225).**

To a solution of **15** in absolute ethanol (400 uL, 0.063 mmol) at room temperature was added **Decane-5,6-dione** (100 mg, 0.587 mmol) and glacial acetic acid (100 uL). The mixture was stirred for 3 hours under nitrogen and then was concentrated *i.v*., The residue was dissolved in CH_2_Cl_2_ (1 mL) and filtered through a glass pipet containing a cotton plug at the neck and about 6 cm of silica gel that had been pre-treated with CH_2_Cl_2_. The material was eluted in sequence using CH_2_Cl_2_ (10 mL), 15% ethanol: 85% CH_2_Cl_2_ (30 mL), and absolute ethanol (5 mL). The collected fractions were analyzed by TLC and those collected during the elution with the ethanol/ CH_2_Cl_2_ mixture were concentrated *i.v.* on the rotovap to give **B1-225** (16 mg, 82% yield) as a yellow-brown oil that formed yellowish crystals upon standing at room temperature in a small amount of CH_2_Cl_2_ (100 uL) overnight. The crystals could be washed with a little ether to remove small amounts of impurities. ^1^H NMR (599 MHz, DMSO- d_6_) δ ppm 0.95 (t, 6 H) 1.30 (t, 3 H) 1.40 - 1.53 (m, 4 H) 1.68 - 1.89 (m, 4 H) 2.85 (br. s., 6 H) 2.91 - 3.10 (m, 4 H) 3.32 – 3.43 (m, 2 H) 3.87 (br. s., 2 H) 4.08 (q, 2 H) 7.11 (s, 1 H) 8.65 (br. s., 1 H) 10.51 (br. s., 1 H). MS: (M + 1)^+^: 417.

**Ethyl N-(2,3-dicyclohexyl-8-[2-dimethylaminoethylamino]-1,4,5-triazanaphthalene-6-yl) carbamate (B1-252P).**

In a procedure similar to that for the synthesis of **B1-225**, to a solution of **15** in absolute ethanol (1.00 mL, 0.16 mmol) at room temperature was added **1,2-Dicyclohexylethanedione** (50 mg, 0.216 mmol) and glacial acetic acid (100 uL). The mixture was stirred for 3 hours at room temperature and then was heated to 60° C for 12 hr under nitrogen. The reaction was then concentrated *i.v*. and the residue was chromatographed using a 4 g silica gel column for MPLC with CH_2_Cl_2_ as the initial eluent. A gradient to 100% of 2: 20: 78:: conc. NH_4_OH: iPrOH: CH_2_Cl_2_) was used. **(B1-252P)** (12 mg, 16% yield) was obtained as a yellow-brown oil. ^1^H NMR (599 MHz, CHLOROFORM-d) δ ppm 1.30 - 1.41 (m, 7 H) 1.41 - 1.55 (m, 4 H) 1.68 - 1.98 (m, 16 H) 2.46 (br. s., 6 H) 2.82 (br. s., 2 H) 2.98 - 3.10 (m, 2 H) 3.57 (br. s., 2 H) 4.26 (q, J=7.22 Hz, 2 H) 7.03 (br. s., 1 H) 7.39 (s, 1 H) 7.82 (br. s., 1 H). MS: (M + 1)^+^: 469.

**Ethyl N-(2,3-dicyclopentyl-8-[2-dimethylaminoethylamino]-1,4,5-triazanaphthalene-6-yl) carbamate (B1-286).**

In a procedure similar to that for the synthesis of **B1-225**, to a solution of **15** in absolute ethanol (1.00 mL, 0.18 mmol, 0.18 M) at room temperature was added **1,2-Dicyclopentylethanedione** (76 mg, 0.36 mmol) and glacial acetic acid (200 uL). The mixture was stirred and heated to 60° C for 4 days under nitrogen. The reaction was then concentrated *i.v*. and the residue was chromatographed using a 4 g silica gel column for MPLC with CH_2_Cl_2_ as the initial eluent. A gradient to 100% of 1: 10: 89:: conc. NH_4_OH: iPrOH: CH_2_Cl_2_) was used. **B1-286** (9 mg, 11% yield) was obtained as a yellow-brown oil. ^1^H NMR (599 MHz, CHLOROFORM-d) δ ppm 1.35 (t, 3 H) 1.62 - 1.81 (m, 8 H) 1.86 - 1.99 (m, 4 H) 2.05 (m, 4 H) 2.42 (br. s., 6 H) 2.72 - 2.84 (m, 2 H) 3.48 - 3.56 (m, 2 H) 3.55 - 3.68 (m, 2 H) 4.25 (q, 2 H) 6.96 (s, 1 H) 7.15 – 7.25 (b. s., 1 H) 7.37 (br. s., 1 H). MS: (M + 1)^+^: 441.

**Ethyl N-(2,3-dihexyl-8-[2-dimethylaminoethylamino]-1,4,5-triazanaphthalene-6-yl) carbamate (B1-250).**

In a procedure similar to that for the synthesis of **B1-225**, to a 0.16 M solution of **15** in methanol (250 uL, 0.0584 mmol) at room temperature was added **Tetradecane-7,8-dione** (23 mg, 0.117 mmol) and glacial acetic acid (100 uL). The mixture was stirred for 3 hours at room temperature and then was heated to reflux for 1 hr. The reaction was then concentrated *i.v*. and the residue was chromatographed using a 4 g silica gel column for MPLC with CH_2_Cl_2_ as the initial eluent. A gradient to 100% of 2: 20: 78:: conc. NH_4_OH: iPrOH: CH_2_Cl_2_) was used. **B1-250** (2 mg, 3% yield) was obtained as a yellow-brown oil. ^1^H NMR (599 MHz, CHLOROFORM-d) δ ppm 0.85 - 0.95 (m, 6 H) 1.28 - 1.40 (m, 9 H) 1.39 - 1.50 (m, 4 H) 1.60 - 1.75 (b. s., 2 H) 1.78 - 1.92 (m, 4 H) 2.33 (s, 6 H) 2.68 (t, J=6.00 Hz, 2 H) 2.93 (t, J=7.61 Hz, 4 H) 3.43 (m, 2 H) 4.26 (q, 2 H) 6.95 (br. s., 1 H) 7.38 (s, 1 H) 7.45 (br. s., 1 H). MS: (M + 1)^+^: 473.

**Ethyl N-(2,3-dioctyl-8-[2-dimethylaminoethylamino]-1,4,5-triazanaphthalene-6-yl) carbamate (B1-257-2).**

In a procedure similar to that for the synthesis of **B1-225**, to a 0.23 M solution of **15** in methanol (250 uL, 0.0.0584 mmol) at room temperature under nitrogen atmosphere was added **Octadecane-9,10-dione**  (33 mg, 0.117 mmol), MeOH (250 uL) and glacial acetic acid (100 uL). The mixture was heated to 65° C briefly to dissolve the diketone and then was stirred for 8 hours at room temperature under nitrogen. The reaction was then concentrated *i.v*. and the residue was chromatographed using a 4 g silica gel column for MPLC with CH_2_Cl_2_ as the initial eluent. A gradient to 100% of 1: 10: 89:: conc. Et_3_N: iPrOH: CH_2_Cl_2_) was used. **B1-257-2** (22 mg, 71% yield) was obtained as a yellow-brown oil. 1H NMR (599 MHz, CHLOROFORM-d) δ ppm 0.81 - 0.94 (m, 6 H) 1.17 - 1.41 (m, 14 H) 1.40 - 1.49 (m, 4 H) 1.84 (dt, J=14.93, 7.47 Hz, 4 H) 2.55 (br. s., 6 H) 2.90 - 2.97 (m, 6 H) 3.59 - 3.77 (m, 2 H) 4.27 (q, J=7.22 Hz, 1 H) 7.07 (br. s., 1 H) 7.40 (s, 1 H) 7.42 – 7.74 (br. s., 1 H). MS: (M + 1)^+^: 529.

**Morpholine-4-yl N’-(2,3-diphenyl-[8-[2-(dimethylamino)ethylamino]-1,4,5-triazanaphthalene-6-yl) urea (B-36).**

In a procedure similar to that for the synthesis of **B1-225**, to a 0.17 M solution of **16** in methanol (0.5 mL, 0.084 mmol) at room temperature under a nitrogen atmosphere was added benzil (1,2-diphenylethanedione; 18 mg, 0.084 mmol) and glacial acetic acid (200 uL). The mixture was stirred for 30 minutes at room temperature and then heated to 65° C under nitrogen for 17 hours. The reaction was then concentrated *i.v*. and the residue was chromatographed using a 12 g silica gel column for MPLC with CH_2_Cl_2_ as the initial eluent. A gradient to 100% of 1: 14: 85:: conc. NH_4_OH: MeOH: CH_2_Cl_2_) was used to elute the product, **B-36** (8 mg, 19% yield), which was obtained as a yellow-brown oil. ^1^H NMR (500 MHz, CHLOROFORM-d) δ ppm 2.33 (s, 6 H) 2.69 (t, J=5.87 Hz, 2 H) 3.47 (br. s., 2 H) 3.63 (br. s., 4 H) 3.76 (br. s., 4 H) 6.96 (br. s., 1 H) 7.28 - 7.41 (m, 7 H) 7.44 - 7.61 (m, 4 H). MS: (M + 1)^+^: 498.

**Piperidine-1-yl N’-(2,3-diphenyl-[8-[2-(dimethylamino)ethylamino]-1,4,5-triazanaphthalene-6-yl) urea (B-48).**

In a procedure similar to that for the synthesis of **B1-225**, to a 0.43 M solution of **17** in iPrOH (0.5 mL, 0.215 mmol) at room temperature under a nitrogen atmosphere was added benzil (1,2-diphenylethanedione; 46 mg, 0.216 mmol) and glacial acetic acid (200 uL). The mixture was stirred for 30 minutes at room temperature and then 1.5 mL more iPrOH was added. The solution was heated to 80° C under nitrogen for 10 hours. The reaction was then concentrated *i.v*. and the residue was chromatographed using a 4 g silica gel column for MPLC with CH_2_Cl_2_ as the initial eluent. A gradient to 100% of 1: 14: 85:: conc. NH_4_OH: MeOH: CH_2_Cl_2_) was used to elute the product, **B-48** (11 mg, 10% yield), which was obtained as a yellowish solid. ^1^H NMR (500 MHz, CHLOROFORM-d) δ ppm 1.57 - 1.73 (m, 6 H) 2.32 (s, 6 H) 2.68 (t, J=6.11 Hz, 2 H) 3.48 (m, 2 H) 3.55 (br. s., 4 H) 6.94 (br. s., 1 H) 7.25 - 7.39 (m, 7 H) 7.51 (d, J=6.85 Hz, 2 H) 7.57 (d, J=7.34 Hz, 2 H) 7.67 (br. s., 1 H). MS: (M + 1)^+^: 496.

**4-Methylpiperazine-1-yl N’-(2,3-diphenyl-[8-[2-(dimethylamino)ethylamino]-1,4,5-triazanaphthalene-6-yl) urea (B-65).**

In a procedure similar to that for the synthesis of **B1-225**, to a 0.23 M solution of **18** in iPrOH (0.5 mL, 0.117 mmol) at room temperature under a nitrogen atmosphere was added benzil (1,2-diphenylethanedione; 25 mg, 0.117 mmol) and glacial acetic acid (200 uL). The mixture was stirred for 30 minutes at room temperature and then 4.5 mL more iPrOH was added. The solution was heated to 80° C under nitrogen for 14 hours. The reaction was then concentrated *i.v*. and the residue was treated with a small amount of conc. NH_4_OH to neutralize the acetate salt of the product. The residue was chromatographed using a 4 g silica gel column for MPLC with CH_2_Cl_2_ as the initial eluent. A gradient to 100% of 1: 14: 85:: conc. NH_4_OH: MeOH: CH_2_Cl_2_) was used to elute the product, **B-65** (12 mg, 20% yield), which was obtained as a yellowish foam. ^1^H NMR (500 MHz, CHLOROFORM-d) δ ppm 2.31 (s, 6 H) 2.34 (s, 3 H) 2.43 - 2.51 (m, 4 H) 2.68 (t, J=6.11 Hz, 2 H) 3.40 - 3.52 (m, 2 H) 3.52 - 3.74 (m, 4 H) 6.94 (br. s., 1 H) 7.24 - 7.38 (m, 7 H) 7.50 (d, J=7.83 Hz, 2 H) 7.52 - 7.57 (m, 2 H) 7.66 (br. s., 1 H). MS: (M + 1)^+^: 511.

**N-Phenyl N’-(2,3-diphenyl-[8-[2-(dimethylamino)ethylamino]-1,4,5-triazanaphthalene-6-yl) urea (B-78).**

In a procedure similar to that for the synthesis of **B1-225**, to a 0.049 M solution of **19** in iPrOH (3.0 mL, 0.15 mmol) at room temperature under a nitrogen atmosphere was added benzil (1,2-diphenylethanedione; 31 mg, 0.15 mmol) and glacial acetic acid (100 uL). The mixture was stirred for 30 minutes at room temperature and then was heated to 80° C under nitrogen for 2 hours. The reaction was then concentrated *i.v*. and the residue was treated with CH_2_Cl_2_ (4.0 mL); the insoluble yellow solid was filtered off and saved. The soluble portion was chromatographed using a 4 g silica gel column for MPLC with CH_2_Cl_2_ as the initial eluent. A gradient to 100% of 1: 14: 85:: conc. NH_4_OH: MeOH: CH_2_Cl_2_) was used to elute the product, and this was combined with the CH_2_Cl_2_-insoluble solid obtained earlier to give **B-78** (combined yield of 58 mg, 78% yield), which was obtained as a yellowish solid. ^1^H NMR (500 MHz, CHLOROFORM-d) δ ppm 2.35 (br. s., 6 H) 2.73 (br. s., 2 H) 3.49 (br. s., 2 H) 6.33 (br. s., 1 H) 6.57 (br. s., 2 H) 6.80 - 6.94 (m, 3 H) 6.97 (br. s., 2 H) 7.06 (br. s., 3 H) 7.29 - 7.96 (m, 8 H). MS: (M + 1)^+^: 504.

**4-(Trifluoromethyl)piperidine-1-yl N’-(2,3-diphenyl-[8-[2-(dimethylamino)ethylamino]-1,4,5-triazanaphthalene-6-yl) urea (B-116).**

In a procedure similar to that for the synthesis of **B1-225**, to a 0.17 M solution of **20** in iPrOH (1.0 mL, 0.169 mmol) at room temperature under a nitrogen atmosphere was added benzil (1,2-diphenylethanedione; 36 mg, 0.169 mmol) and glacial acetic acid (100 uL). The mixture was stirred for 30 minutes at room temperature and then the solution was heated to 90° C under nitrogen for 2 hours. The reaction was then concentrated *i.v*. and the residue was chromatographed using a 4 g silica gel column for MPLC with CH_2_Cl_2_ as the initial eluent. A gradient to 100% of 2: 20: 78:: conc. NH_4_OH: MeOH: CH_2_Cl_2_) was used to elute the product, **B-116** (51 mg, 54% yield), which was obtained as a yellowish foam. ^1^H NMR (500 MHz, CHLOROFORM-d) δ ppm 1.56 - 1.69 (m, 2 H) 1.93 - 2.02 (m, 2 H) 2.22 - 2.31 (m, 1 H) 2.33 (s, 6 H) 2.69 (t, J=6.11 Hz, 2 H) 2.83 - 3.01 (m, 2 H) 3.41 - 3.54 (m, 2 H) 4.42 (br. s., 1 H) 6.97 (br. s., 1 H) 7.28 (s, 1 H) 7.28 - 7.39 (m, 6 H) 7.47 - 7.60 (m, 4 H). MS: (M + 1)^+^: 564.

**4,4-Dimethylpiperidine-1-yl N’-(2,3-diphenyl-[8-[2-(dimethylamino)ethylamino]-1,4,5-triazanaphthalene-6-yl) urea (B-128).**

In a procedure similar to that for the synthesis of **B1-225**, to a 0.08 M solution of **21** in 95% ethanol (2.5 mL, 0.20 mmol) at room temperature under a nitrogen atmosphere was added benzil (1,2-diphenylethanedione; 50 mg, 0.24 mmol) and glacial acetic acid (500 uL). The mixture was stirred for 3 days at room temperature and then the solution was heated to 80° C under nitrogen for 2 hours. The reaction was then concentrated *i.v*. and the residue was chromatographed using a 4 g silica gel column for MPLC with CH_2_Cl_2_ as the initial eluent. A gradient to 100% of 2: 20: 78:: conc. NH_4_OH: MeOH: CH_2_Cl_2_) was used to elute the product, **B-128** (62 mg, 58% yield), which was obtained as a yellow powder. ^1^H NMR (500 MHz, CHLOROFORM-d) δ ppm 1.02 (s, 6 H) 1.38 - 1.52 (m, 4 H) 2.33 (s, 6 H) 2.71 (t, J=6.11 Hz, 2 H) 3.45 - 3.53 (m, 2 H) 3.57 (br. s., 4 H) 6.99 (br. s., 1 H) 7.24 - 7.38 (m, 7 H) 7.51 (d, J=6.85 Hz, 2 H) 7.56 (d, J=7.34 Hz, 2 H) 7.64 (br. s., 1 H). MS: (M + 1)^+^: 524.

**3,5-Dimethyl-piperidine-1-yl N’-(2,3-diphenyl-[8-[2-(dimethylamino)ethylamino]-1,4,5-triazanaphthalene-6-yl) urea (B-160).**

In a procedure similar to that for the synthesis of **B1-225**, to a 0.11 M solution of **22** in iPrOH (1.3 mL, 0.15 mmol) at room temperature under a nitrogen atmosphere was added benzil (1,2-diphenylethanedione; 50 mg, 0.24 mmol) and glacial acetic acid (100 uL). The mixture was stirred for 30 minutes at room temperature and then the solution was heated to 80° C under nitrogen for 3 hours. The reaction was then concentrated *i.v*. and the residue was chromatographed using a 4 g silica gel column for MPLC with CH_2_Cl_2_ as the initial eluent. A gradient to 100% of 2: 20: 78:: conc. NH_4_OH: MeOH: CH_2_Cl_2_) was used to elute the product, **B1-160** (44 mg, 58% yield), which was obtained as a yellow glass. ^1^H NMR (500 MHz, CHLOROFORM-d) δ ppm 0.73 - 0.86 (m, 2 H) 0.94 (d, J=6.36 Hz, 6 H) 1.46 - 1.55 (m, 1 H) 1.83 - 1.92 (m, 1 H) 2.32 (s, 6 H) 2.36 - 2.47 (m, 2 H) 2.65 - 2.74 (m, 2 H) 3.43 - 3.52 (m, 2 H) 4.12 (m, 2 H) 6.93 (br. s., 1 H) 7.28 - 7.39 (m, 7 H) 7.49 - 7.53 (m, 2 H) 7.54 - 7.61 (m, 2 H) 7.65 (br. s., 1 H). MS: (M + 1)^+^: 524.

**Piperidine-1-yl N’-(2,3-diphenyl-[8-(4-methylpiperazin-1-yl)-]-1,4,5-triazanaphthalene-6-yl) urea (B-136).**

In a procedure similar to that for the synthesis of **B1-225**, to a 0.052 M solution of **23** in iPrOH (4.0 mL, 0.207 mmol) at room temperature under a nitrogen atmosphere was added benzil (1,2-diphenylethanedione; 50 mg, 0.24 mmol) and glacial acetic acid (500 uL). The mixture was stirred for 30 minutes at room temperature and then the solution was heated to 80° C under nitrogen for 5 hours. The reaction was then concentrated *i.v*. and the residue was chromatographed using a 4 g silica gel column for MPLC with CH_2_Cl_2_ as the initial eluent. A gradient to 100% of 2: 20: 78:: conc. NH_4_OH: MeOH: CH_2_Cl_2_) was used to elute the product, **B-136**(23 mg, 22% yield), which was obtained as a yellow glass. ^1^H NMR (500 MHz, CHLOROFORM-d) δ ppm 1.65 (br. s., 6 H) 2.40 (s, 3 H) 2.71 (br. s., 4 H) 3.54 (br. s., 4 H) 3.92 (br. s., 4 H) 7.28 - 7.39 (m, 7 H) 7.51 (d, J=6.85 Hz, 2 H) 7.63 (d, J=7.34 Hz, 2 H) 7.76 (br. s., 1 H) 7.96 (br. s., 2 H). MS: (M + 1)^+^: 508.

**4-Trifluoromethylpiperidine-1-yl N’-(2,3-diphenyl-[8-(4-methylpiperazin-1-yl)-]-1,4,5-triazanaphthalene-6-yl) urea (B-141).**

In a procedure similar to that for the synthesis of **B1-225**, to a 0.046 M solution of **24** in iPrOH (4.0 mL, 0.185 mmol) at room temperature under a nitrogen atmosphere was added benzil (1,2-diphenylethanedione; 80 mg, 0.38 mmol) and glacial acetic acid (800 uL). The mixture was stirred for 30 minutes at room temperature and then the solution was heated to 80° C under nitrogen for 3 hours. The reaction was then concentrated *i.v*. and the residue was chromatographed using a 4 g silica gel column for MPLC with CH_2_Cl_2_ as the initial eluent. A gradient to 100% of 1: 10: 89:: conc. NH_4_OH: MeOH: CH_2_Cl_2_) was used to elute the product, **B-141** (74 mg, 69% yield), which was obtained as a yellow glass. ^1^H NMR (500 MHz, CHLOROFORM-d) δ ppm 1.51 - 1.69 (m, 2 H) 1.97 (d, J=12.72 Hz, 2 H) 2.20 - 2.35 (m, 1 H) 2.40 (s, 3 H) 2.65 - 2.77 (m, 4 H) 2.93 (br. s., 2 H) 3.87 (br. s., 4 H) 4.30 (br. s., 2 H) 7.28 - 7.40 (m, 7 H) 7.50 (d, J=6.85 Hz, 2 H) 7.61 (br. s., 2 H) 7.91 (br. s., 1 H). MS: (M + 1)^+^: 576.

**4-Trifluoromethylpiperidine-1-yl N’-(2,3-diphenyl-[8-[4-[2-(dimethylamino)ethyl]piperazin-1-yl]-1,4,5-triazanaphthalene-6-yl) urea (B-152).**

In a procedure similar to that for the synthesis of **B1-225**, to a 0.041 M solution of **25** in iPrOH (4.0 mL, 0.164 mmol) at room temperature under a nitrogen atmosphere was added benzil (1,2-diphenylethanedione; 80 mg, 0.38 mmol) and glacial acetic acid (800 uL). The mixture was stirred for 30 minutes at room temperature and then the solution was heated to 80° C under nitrogen for 2 hours. The reaction was then concentrated *i.v*. and the residue was chromatographed using a 4 g silica gel column for MPLC with CH_2_Cl_2_ as the initial eluent. A gradient to 100% of 1: 10: 89:: conc. NH_4_OH: MeOH: CH_2_Cl_2_) was used to elute the product, **B-152** (79 mg, 76% yield), which was obtained as a yellow glass. 1H NMR (500 MHz, CHLOROFORM-d) δ ppm 1.54 - 1.70 (m, 2 H) 1.94 - 2.03 (m, 2 H) 2.23 - 2.34 (m, 1 H) 2.38 (br. s., 6 H) 2.57 - 2.69 (m, 4 H) 2.73 - 2.83 (m, 4 H) 2.90 - 3.01 (m, 2 H) 3.85 - 3.99 (m, 4 H) 4.24 - 4.35 (m, 2 H) 7.29 – 7.39 (m, 7 H) 7.46 - 7.54 (m, 2 H) 7.58 - 7.66 (m, 2 H) 7.90 (br. s., 1 H). MS: (M + 1)^+^: 633.
